# The Cytoscape BioGateway App: explorative network building from an RDF store

**DOI:** 10.1093/bioinformatics/btz835

**Published:** 2019-11-09

**Authors:** Stian Holmås, Rafel Riudavets Puig, Marcio Luis Acencio, Vladimir Mironov, Martin Kuiper

**Affiliations:** 1 Semantic Systems Biology Group, Department of Biology; 2 Department of Clinical and Molecular Medicine, Norwegian University of Science and Technology, 7491 Trondheim, Norway

## Abstract

**Summary:**

The BioGateway App is a Cytoscape (version 3) plugin designed to provide easy query access to the BioGateway Resource Description Framework triple store, which contains functional and interaction information for proteins from several curated resources. For explorative network building, we have added a comprehensive dataset with regulatory relationships of mammalian DNA-binding transcription factors and their target genes, compiled both from curated resources and from a text mining effort. Query results are visualized using the inherent flexibility of the Cytoscape framework, and network links can be checked against curated database records or against the original publication.

**Availability and implementation:**

Install through the Cytoscape application manager or visit www.biogateway.eu for download and tutorial documents.

**Supplementary information:**

Supplementary information is available at *Bioinformatics* online.

## 1 Introduction

Semantic web technologies provide a powerful framework for the integration of diverse datasets into one homogeneous, queryable resource. Many primary life science databases make their content available as RDF (Lassila *et al.*, 1998) graphs, through triple store endpoints, including UniProtKB (UniProt Consortium, 2017, sparql.uniprot.org) and IntAct (Orchard *et al.*, 2014) and Reactome (Fabregat *et al.*, 2018; www.ebi.ac.uk/rdf/datasets/). In addition, resources like Bio2RDF (Callahan *et al.*, 2013, bio2rdf.org/), Pathway Commons (Cerami *et al.*, 2011, rdf.pathwaycommons.org/) and BioGateway (Antezana *et al.*, 2009, www.biogateway.eu) provide RDF with content integrated from several public resources.

The BioGateway knowledge base (Antezana *et al.*, 2009) was one of the first semantic web resources developed to service the domain of the Life Sciences. Version BioGateway 2.1 hosts RDF data for ∼1500 proteomes (species) stored as graphs (http://biogw-db.nt.ntnu.no:15990/sparql), as opposed to the more common relational databases. Version BioGateway 3.0 (www.biogateway.eu) contains mainly human proteome-centric data, which can be queried with the SPARQL query language (Prud’hommeaux and Seaborne, 2008). Powerful as this query language is for speed and query complexity, designing SPARQL queries tends to be rather intimidating to the average biologists, including many who would be interested in network assembly from available knowledge sources. Therefore, the wealth of information that triple stores provide has only been fully used by experts able to build their own SPARQL queries. To improve ease of access, we reach out to the large user base of the Cytoscape platform (Cline *et al.*, 2007) and make the BioGateway content available for exploratory network building through a Cytoscape plugin: the BioGateway App. This allows users to take advantage of the inherent advantages of a graph-based database while querying and assessing the results as graphs in the familiar setting of the Cytoscape network editor, further aided by its extensive functionality for network analysis.

## 2 Description of the BioGateway App

**Fig 1.**
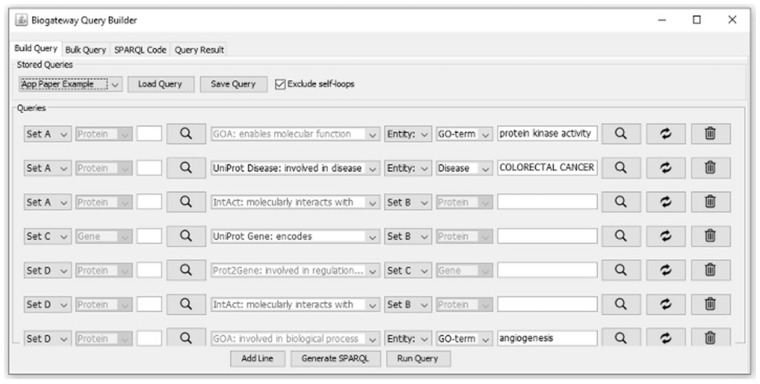
Screenshot of an example query in the BioGateway App Query Builder

The main feature of the BioGateway App is the *Query Builder* (see example in Fig. 1), which supports the design of queries that are built from definitions of proteins or genes and a relationship to either an ontology term or another protein or gene. By adding additional query parts line by line (Fig. 1), increasingly complex and restrictive or inclusive queries can be composed. The *Run Query* command converts these to native SPARQL queries that are launched against the BioGateway 3.0 SPARQL endpoint (www.biogateway.eu/sparql-endpoint/). The example in Figure 1 is further explained in Supplementary Material S1, and tutorial pages are provided in Supplementary Material S2. All materials can also be viewed at www.biogateway.eu/app/.

The main information that is subject to the query is obtained from IntAct (protein–protein interactions; Orchard *et al.*, 2014), UniProtKB (protein descriptions, their genes, related diseases; UniProt Consortium, 2017) and the Gene Ontology database (protein annotations; Gene Ontology Consortium, 2018). To allow a user a special focus on gene regulation, we also included several resources with regulatory relations (transcription factor–target gene) of transcription factors (TF) with one or more target genes (TG): TFactS (Essaghir *et al.*, 2010), TRRUST (Han *et al.*, 2015), IntAct (Orchard *et al.*, 2014), Signor (Perfetto *et al.*, 2016), HTRIdb (Bovolenta *et al.*, 2012) and GOA (Gene Ontology Consortium, 2018). In addition to these curated resources, we have added a resource produced by a text mining effort, named EXTRI (www.extri.org).

The selection of terms of interest, such as genes, proteins, ontology terms and relation types is facilitated by an autocomplete function that is driven by a REST API of a NoSQL database loaded with all the entity names and metadata from the BioGateway server, to allow quick response times. To ensure compatibility between the App and the BioGateway data, upon startup the App fetches an XML-based configuration file from the NoSQL server. This file contains the relation types and their URIs, default settings and the default layout style for BioGateway graphs, as well as available metadata types and the query constraints that this metadata enables, allowing some updates to the App without requiring the user to reinstall it. User preferences set in the BioGateway tab of the Cytoscape Control Panel are stored between sessions, such as the default query constraints related to species, data sources and additional selection criteria for querying the BioGateway content.

Next to specifying the results through the definition of restrictive queries, the *Query Constraints* section of the BioGateway tab in the Control Panel allows additional constraints for specific relations, such as setting a minimum confidence score for Protein–Protein Interactions, as provided by IntAct (Orchard *et al.*, 2014). This control panel also allows the selection of extra types of metadata to be loaded together with the results, but as this may significantly increase query time, this metadata can also be added after the network is complete (*Reload Metadata*), so that it can be used for filtering and display options.

## 3 Conclusion and future work

The BioGateway App provides a versatile, user-friendly query interface to the BioGateway triple store. By extracting some of the BioGateway content to a NoSQL database, we have been able to combine the power of graph queries with the speed of interactive querying, and also implemented a mechanism to synchronize and update the App with respect to data changes in BioGateway. BioGateway covers several of the Elixir Core Resources, and we believe that the App adds significant versatility and user friendliness to their use. Even though by the time of this manuscript submission only human data had been loaded into BioGateway 3.0, in the near future its taxonomic coverage will grow to ∼25 proteomes. With the EXTRI results obtained through text mining, the coverage of potential TF–TG interactions is much more comprehensive. Interestingly, this could also set the stage for ‘community curation’: potentially important network links could be checked on their source pages through interfaces that will invite a user to manually curate these links, e.g. through Europe PMC’s SciLite annotation tool [Venkatesan *et al.*, 2016; see also Use Case 2, tutorial document (Supplementary Material S2)]. The results of such a curation action could easily be incorporated into the BioGateway NoSQL metadata through an API. The EXTRI resource will soon be updated with higher quality results (www.extri.org), and these results will also be made available in the SciLite platform.

Because of the need for intuitive, fast explorative querying that a user would expect from the App, the RDF data model of BioGateway necessarily underwent several cycles of optimization in response to user feedback and additional requests (to be published elsewhere, for now: see data model, www.biogateway.eu/#datamodel). While the intuitive user-friendly layout of the Query Builder inevitably restricted to some extent the range of possible SPARQL queries, the changed data model continues to support the full power of direct queries through our SPARQL endpoint.

## Supplementary Material

btz835_Supplementary_Data
