# Supplementary material for: The Cytoscape BioGateway App: explorative network building from an RDF store
Source: Bioinformatics. 2019 Nov 9;36(6):1966–7. doi: 10.1093/bioinformatics/btz835 (PMC7703768; doi:10.1093/bioinformatics/btz835)
Supplement: btz835_Supplementary_Data [file btz835_supplementary_data.zip › btz835-Suppl_Data/Bgw_SupMat_documentationS2.pdf]

S2. BioGateway App Documentation

Getting Started **3**
Installation 3
Querying Networks 4
Building Queries 4
Example Tutorial 6
Allowing Self-loops in the network 13
Reproducing the example from the paper 14

Example Use Cases **18**
Use Case 1 - Exploring Around a Node 19
Use Case 2 - Building a TF-TG network starting from a TF of interest 22
Use case 3 - Establishing Molecular Relationships between Diseases 26
Use case 4 - Find the Proteins connecting two GO Terms 27
Use case 5 - Find proteins with protein kinase activity involved in a disease and the context around them 28
Use case 5 (Extension) - Generalise a network from BioGateway for further analysis with Cytoscape apps 30
Use case 6 - Connect Protein A to Protein B (with GO restriction C) 31
Use case 7 - Potential downstream effects after targeted inhibition of proteins 33

Application Manual **35**
Create your own query 35
Creating Advanced Queries 36
The Exclude self-loops Setting 37
Exclude Self-loops option enabled 37
Exclude Self-loops option disabled 38
Saving/Loading queries 38
Saving queries 39
Loading queries 39
The SPARQL behind the scene 41
Query Builder Results 42
Filter results based on nodes in selected network 42
Only relations TO nodes in current network 42
Only relations FROM nodes in current network 43
Text Filtering 43
Select Paths 43
Reviewing relations 46
Going to the source of the data 46
Going to PubMed articles 47
Going to ‘Landing Pages’ 47
Metadata loading 48
Bulk Query 49

Right Click Queries 50
Adding nodes 50
Running queries on the nodes in the network 51
Find common relations FROM/TO selected 53
Selecting/deselecting active properties in the Control Panel 54
The BioGateway Layout Style 55
Nodes Layout 56
Edges Layout 56
Changing the Font Size in the Query Builder 57

Getting Started

Installation

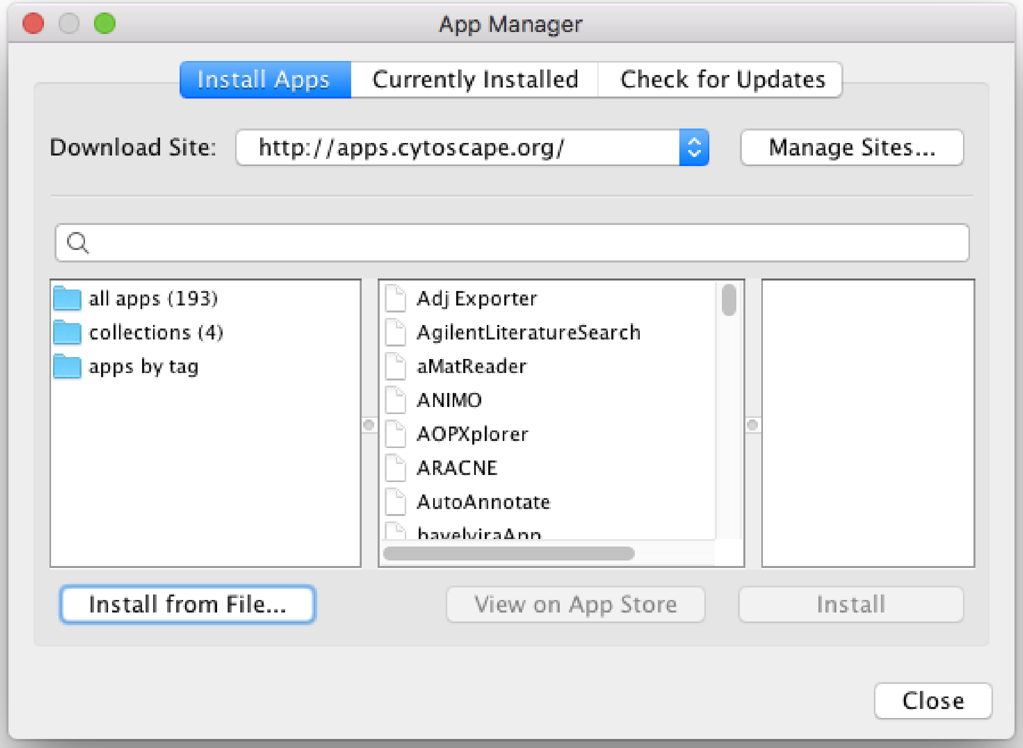

To install the App, use the App Manager in Cytoscape (version 3.7.0 or later), click “Install from File…”, and select the downloaded file.

Querying Networks

Building Queries

BioGateway is an RDF Graph Database or 'triple store', and can be searched by building a query consisting of a set of questions that together specify what you are looking for. In this context, the "graph" is a network of nodes – shown as circles – and edges – the arrows connecting the nodes (see Example Graph Database, animation, to the right).

In the animation to the right, we see a representation of a mock network in a (very small) graph database of animals and some of their properties. The first question (part of a final query) selects for the animals that have the property of being "kept as" a "Pet". This returns a subset of the network containing the nodes representing "Pet", and the animals with edges of the type "Kept as" pointing to the "Pet" node.

By adding more question parts to the query, we can further specify the search. The second line in the query restricts the pets to those that are "Walking", eliminating the Parrot from the results. And by further constraining the results to those pets that also are "Chasing" mice, we end up with a final query that results in the network shown in the last part of the animation.

Note that even though "Mouse" is not an animal satisfying all the conditions of the query, it is included because it is part of the relevant network for the query.

The BioGateway database is powered by Virtuoso, and queryable through SPARQL, a graph query language based on the same principle as above. We construct a query by step-wise specifying the nodes we want, and the relationships / type of edges between them.

Each new line represents an additional part of the query, consisting of a subject, predicate and object. Subjects and objects are always nodes, while predicates are edges. In BioGateway, these edges are also called relation types, because they represent a type of relation between two entities.

In BioGateway, the subjects and objects can either be bound to a specific value, like the node representing "Pet" or "Walking" in the animation above, or they can represent any value satisfying the conditions of the query. In the Cytoscape BioGateway App these unbound values are called "Sets", as they represent the set of all values qualifying their part of the query.

The last query from the animation above could be formulated as a BioGateway query formulated as:

```
?animal kept_as Pet

?animal moves_by Walking

?animal chases Mouse
```

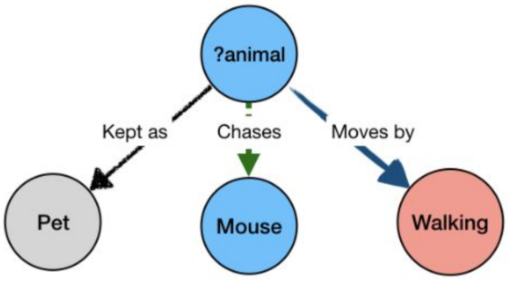

The "?animal" part is not bound to a specific value, but rather any value that can simultaneously satisfy all the parts of the query – i.e. be kept as a pet, and move by walking, and chase mice. The result of the query would be:

```
Cat    kept_as    Pet
Cat    moves_by   Walking
Cat    chases     Mouse
```

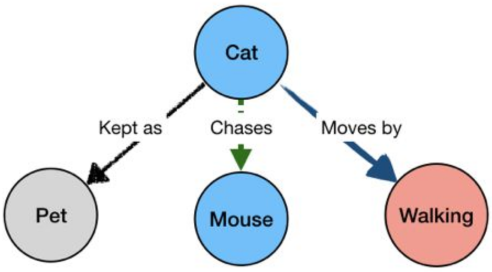

This is because "Cat" is the only value of "?animal" that would satisfy all the constraints of our query. For a simpler query, like the initial one from the animated example:

```
?animal kept_as Pet
```

We would get all the matching pets as results:

```
Cat kept_as Pet
Mouse kept_as Pet
Parrot kept_as Pet
Dog kept_as Pet
```

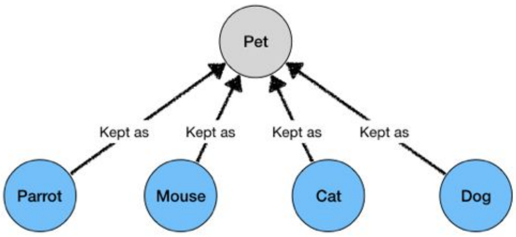

Example Tutorial

In the BioGateway App, we can use the Query Builder to build queries in a step-wise manner, using the same format as described above. A query consists of one or more lines, each specifying a selection of either a specific node or set of nodes, and their relation with another specific node/ set of nodes.
To see how this works, open the query builder, and load “Example 1a”.

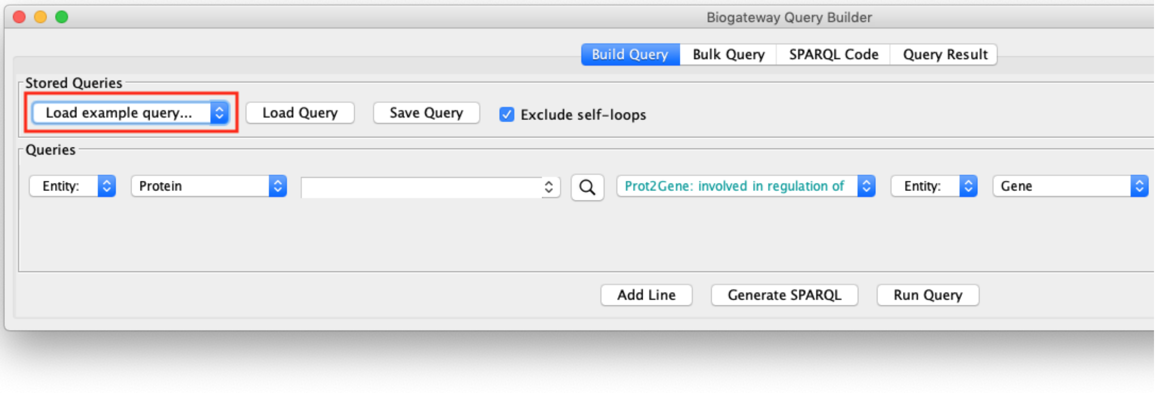

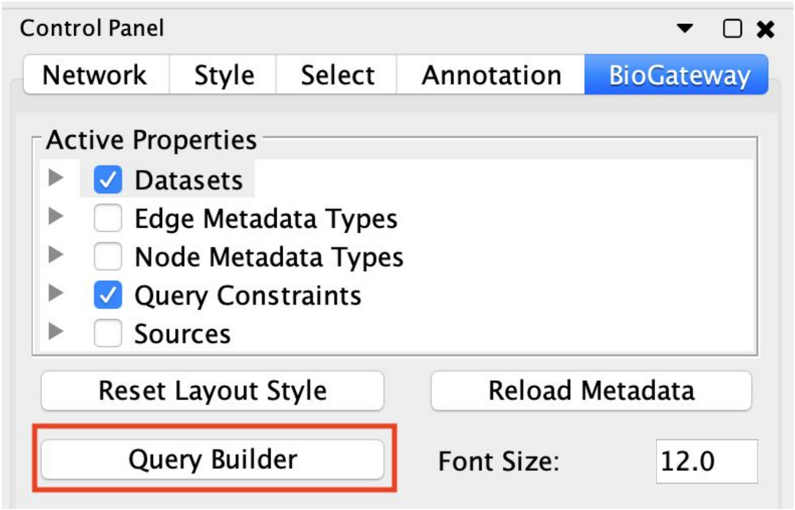

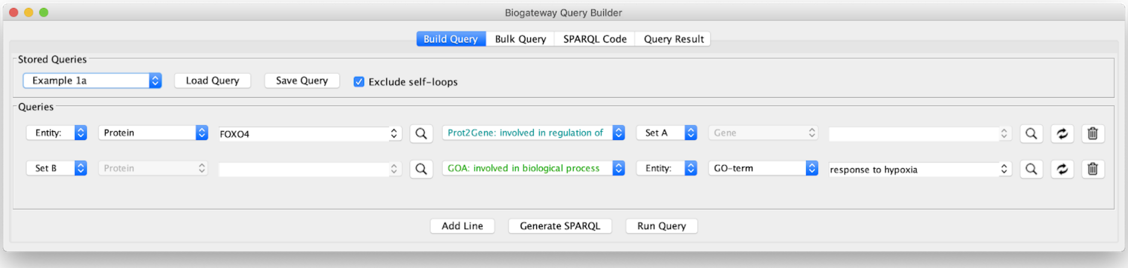

The “Example 1a” query shows two lines that specify and return: (1) all the genes that are a transcriptional target of the transcription factor protein FOXO4; and (2) all the proteins that are annotated to be involved in the GO Biological Process “response to hypoxia”. Clicking on the Run Query button will launch the query against the BioGateway backend and open the Query Results tab. For all the examples in this section we will select all the results (click in

results table and press CtrA) and import them to a new network by clicking on “Import to New Network”.

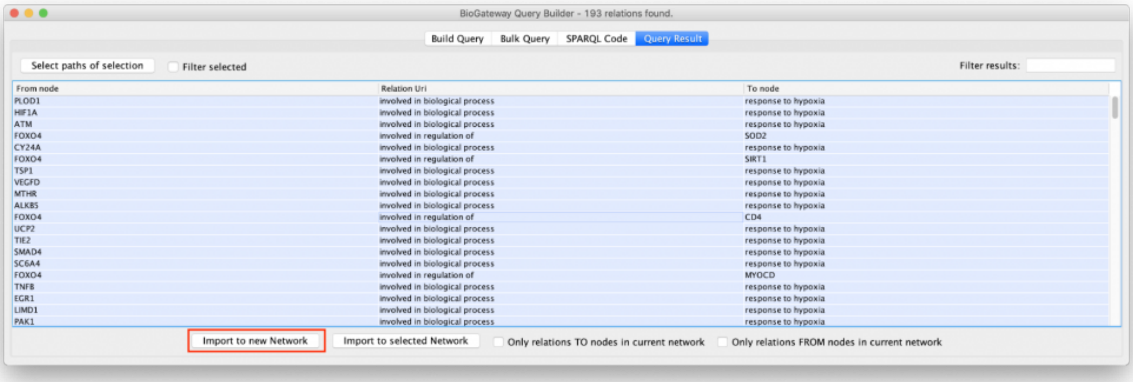

Note that the result of this query contains two separate clusters of nodes. There are two reasons for that:

1. Lines 1 and 2 are asking for two independent and different sets (Set A in line 1, and Set B in line 2).
2. Set A is composed of genes, while Set B is composed of proteins. It is important to bear in mind that Genes and Proteins are different entities in BioGateway.

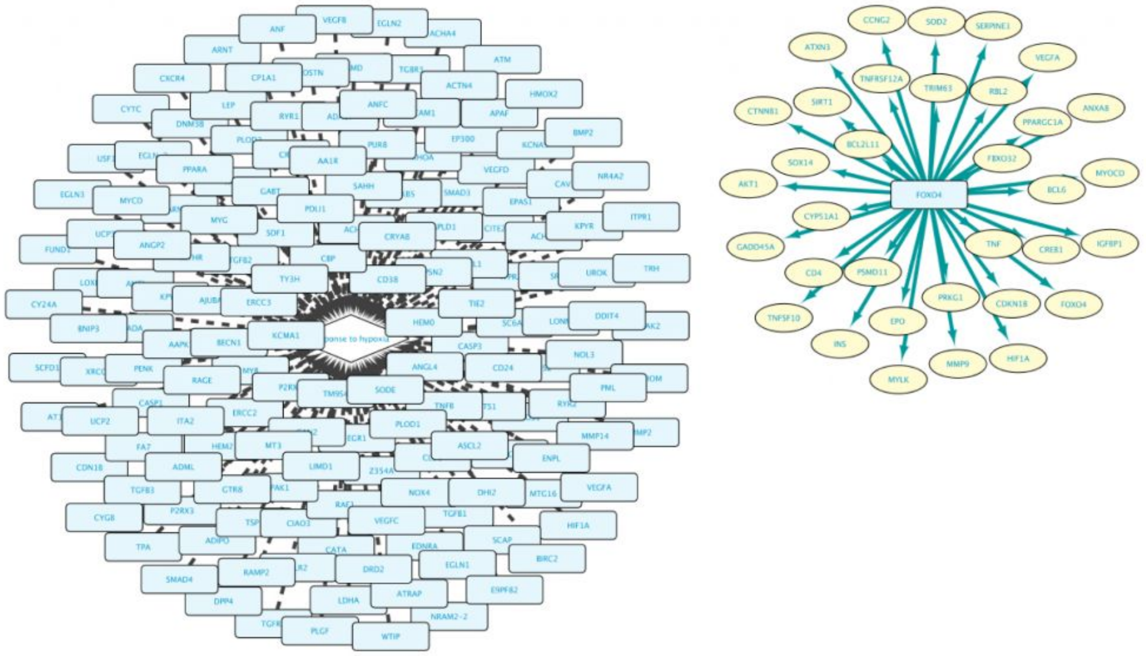

Let’s include an extra specification in the query as shown in “Example 1b”. Clicking on that query shows a third specification:

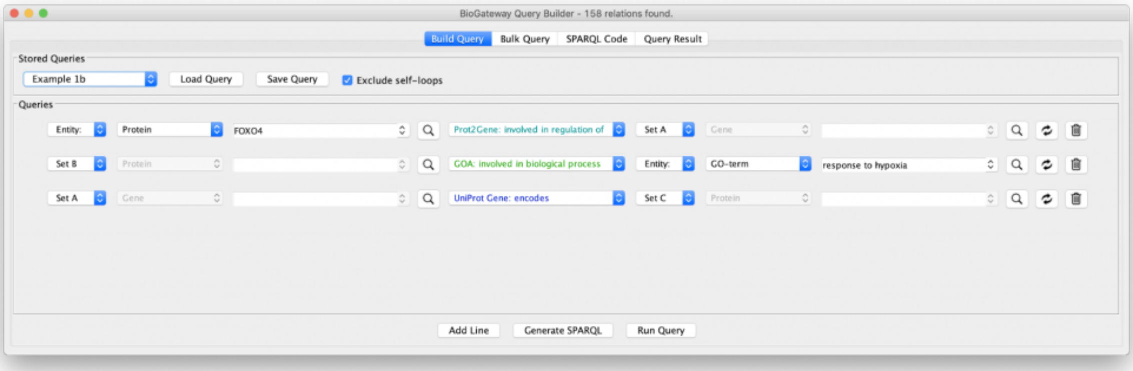

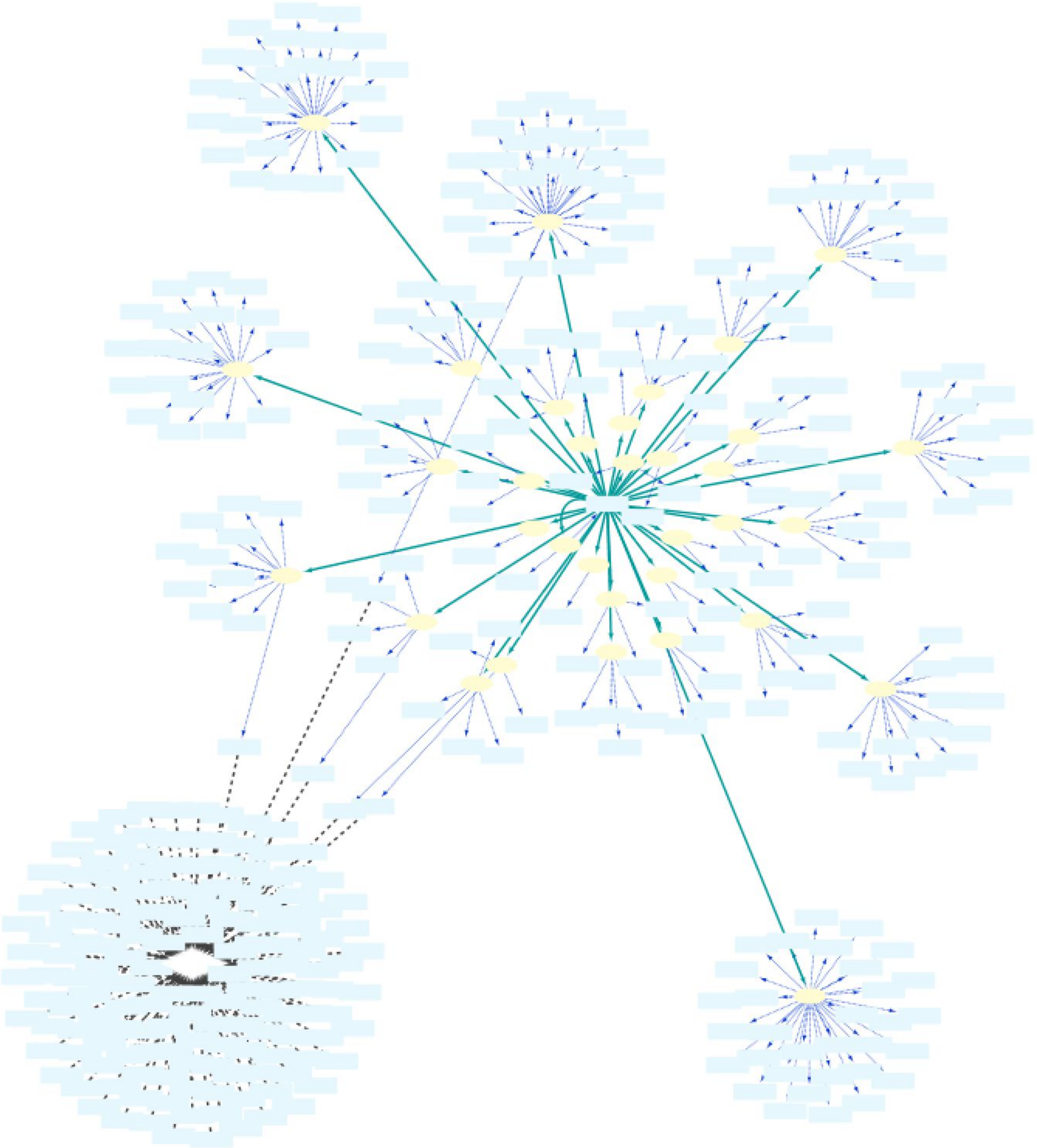

The new line asks for all the proteins (Set C) being encoded by the genes in Set A. The

results of this query shows an even larger network with many of the genes now functioning as hubs connected to many proteins, because of the fact that a gene can code for many proteins. In addition, the network is now fully connected, because some of the genes code for proteins that are annotated with the GO term selected in query line 2.
To obtain a smaller network, in "Example 1c" the Set C in line 3 is redefined to Set B. This small difference now restricts the proteins encoded by Set A to those that are involved in the Biological Process "response to hypoxia":

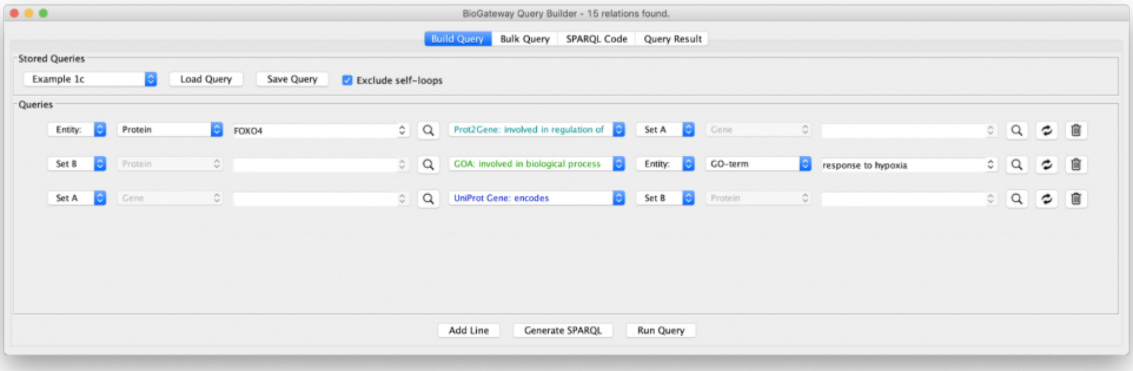

The resulting network shown below is now much more manageable, and to the point. (We used Cytoscape's yFiles hierarchic layout algorithm for this figure.)

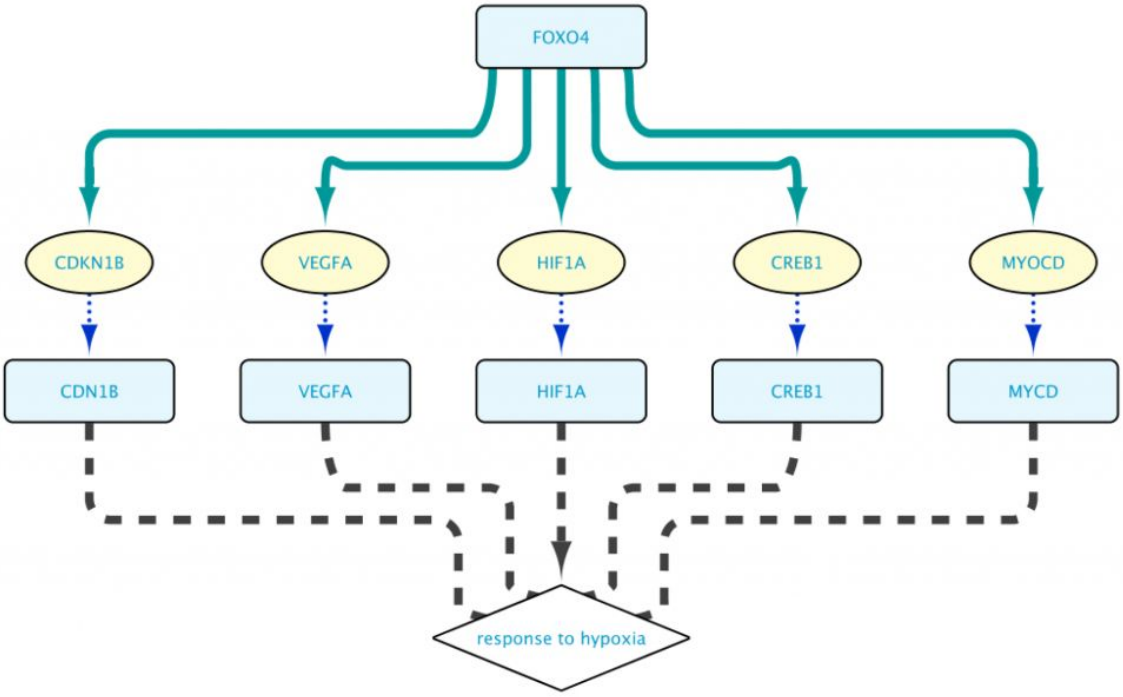

This network can be further extended by adding an additional query line, as shown in the "Example 1d" query. Line 4 specifies the following: return all the proteins (Set C) that interact with the proteins that are encoded by genes regulated by FOXO4 (Set B).

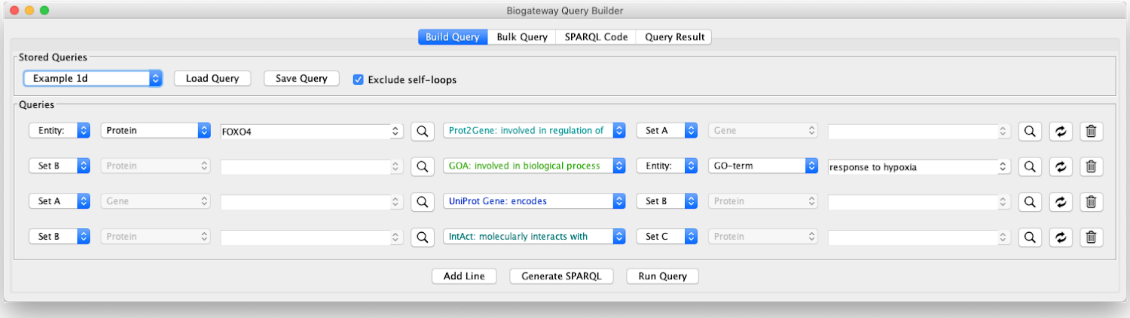
Biogateway Query Builder
Build Query
Bulk Query
SPARQL Code
Query Result
Stored Queries
Example 1d
Load Query
Save Query
Exclude self-loops
Queries
Entity:
Protein
FOXO4
Prot2Gene: involved in regulation of
Set A
Gene
Set B
Protein
GOA: involved in biological process
Entity:
GO-term
response to hypoxia
Set A
Gene
UniProt Gene: encodes
Set B
Protein
Set B
Protein
IntAct: molecularly interacts with
Set C
Protein
Add Line
Generate SPARQL
Run Query

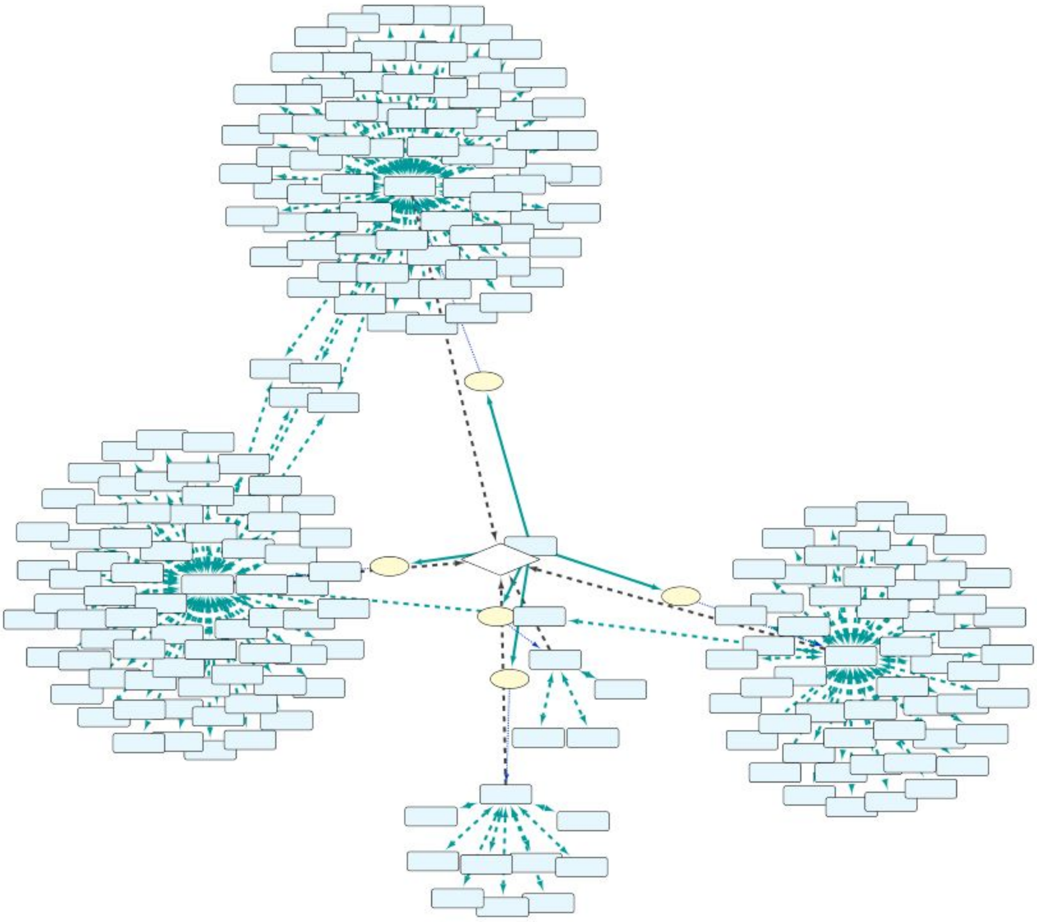

As can be seen in the image below, by adding an extra selection to the query we have increased again the size of the network. One option to reduce the size again is shown in “Example 1e”, where the interacting proteins in Set C are restricted to those involved in the Biological Process “response to hypoxia”.

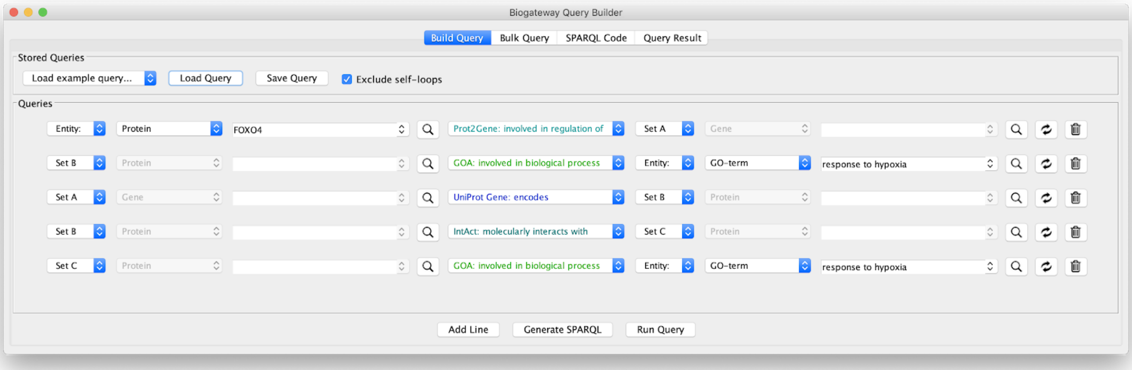

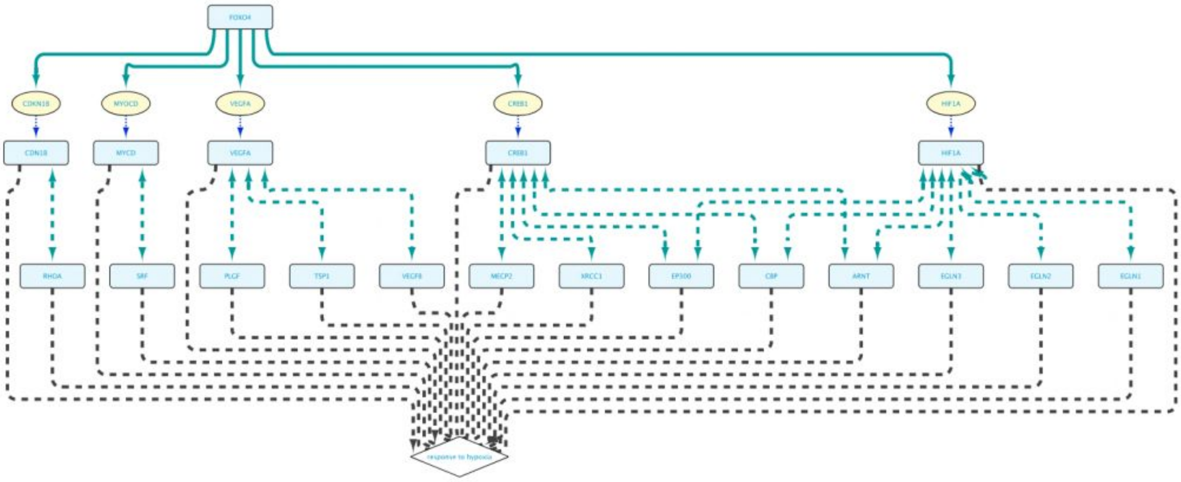

The real value in the Query Builder is the ability to use it to formulate your own queries. An important help in this is the Autocomplete Search feature, which will help you to find the correct biological entities and relationships that you are interested in for your query.

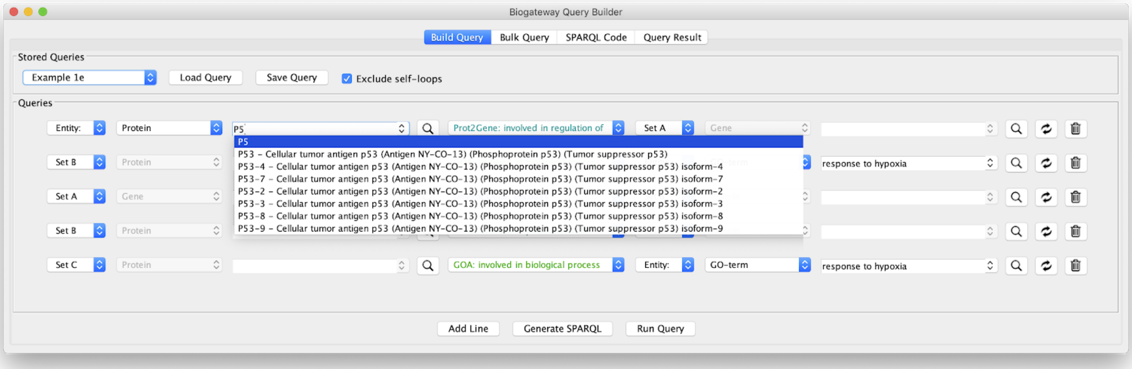

With the help of the autocomplete search function you can quickly and efficiently redefine

any of the biological entities or the relationships from a query. The figure below shows the results after replacing FOXO4 with MYC in line 1, and the GO term “response to hypoxia” with ‘response to ionizing radiation” (line 2). This illustrates that all the examples from 1a to 1e can be used as templates for your own queries, and you will appreciate better the versatility of the App when tweaking these queries.

The next step is to learn how to create your own queries from scratch. For an introduction to that, please continue to the App Manual. More examples on how to use the BioGateway App to tackle Biological questions can also be found in the Use Cases page.

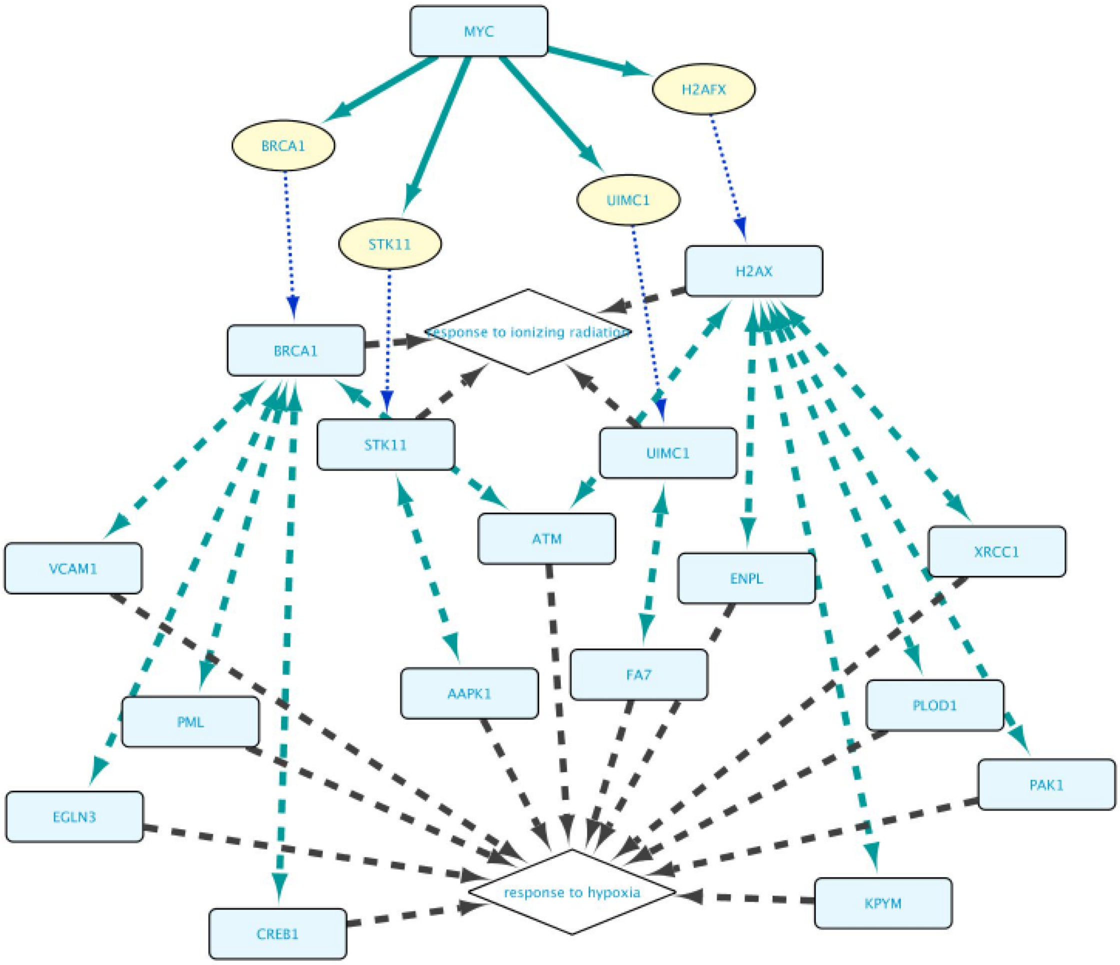

Allowing Self-loops in the network

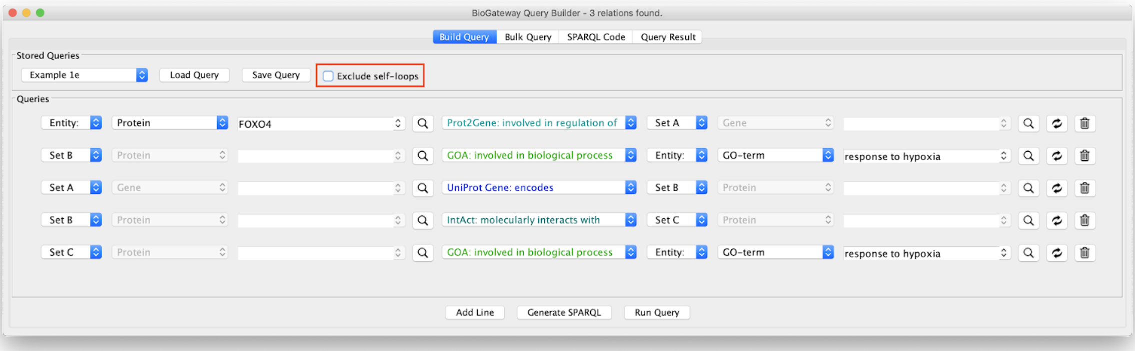

The Exclude self-loops option in the Query Builder is by default activated. Queries can be run with or without including self loops, meaning results that stem from entities having relationships with itself, for instance a protein having a homodimer relationship. Including self loops often results in (many) more results.

The effect of this option is illustrated in the figure to below: load the Example 1e query in the Query Builder followed by unchecking the Exclude self-loops checkbox. This will now allow the network to contain self interactions of the nodes.

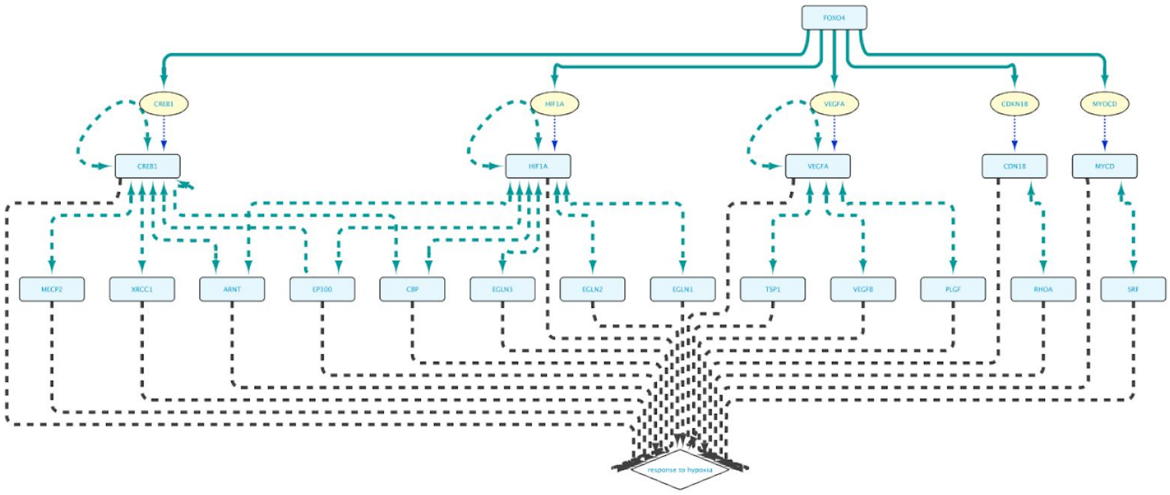

Next, run the query and importing all the results shows a network contains several self-loops of proteins. A more detailed explanation of this Setting can be found in the [App Manual.](#)

Reproducing the example from the paper

This section is dedicated to manually reproduce the results from the paper. The first step to do so will be to open the Query Builder.

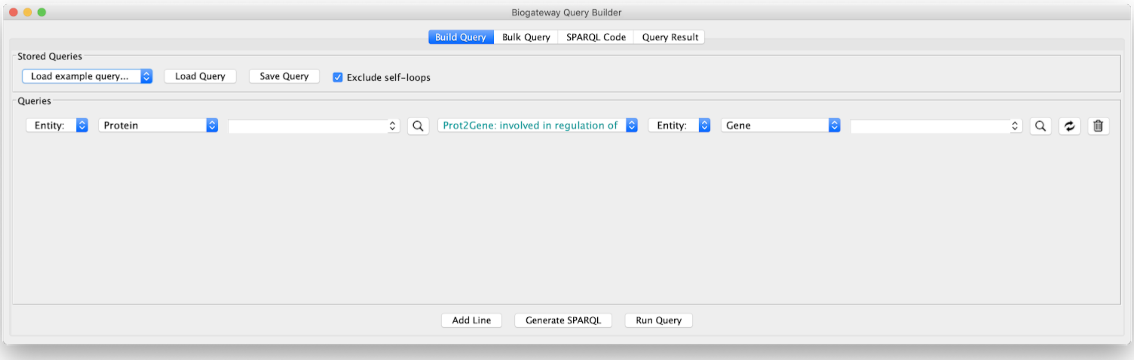

Next, we will create query lines one by one; after completing one the next line can be added by clicking the Add Line button:

- Line 1: set the Subject to Set A, the relation type to GOA: enables molecular function, and the Object to Entity. Start typing protein kinase activity in the text field corresponding to the Object until the autocomplete search engine displays the GO term. Once found, select it by clicking.
- Line 2: set the Subject again to Set A to restrict all proteins involved in Colorectal Cancer to only those with protein kinase activity. The relation type will be UniProt Disease: involved in disease. Finally, the Object will be set to Entity, and the text field will have to contain COLORECTAL CANCER. You will see that there are several hits for Colorectal Cancer. To ensure the reproducibility of this example, select the one written completely in upper case.
- Line 3: set the Subject to Set A, the relation type to Intact: molecularly interacts with, and the Object to Set B. This will find all PPIs containing the proteins found in lines 1 and 2.
- Line 4: set the Subject to Set C, the relation type to UniProt Gene: encodes, and the Object to Set B.
- Line 5: set the Subject to Set D, the relation type to Prot2Gene: involved in regulation of, and the Object to Set C.
- Line 6: set the Subject to Set D, the relation type to Intact: molecularly interacts with, and the Object to Set B.
- Line 7: set the Subject to Set D, the relation type to GOA: involved in biological process. Set the Object to Entity and write angiogenesis in the corresponding text field until the autocomplete engine finds the term. Select it by clicking on it.

The final query should look like the image below:

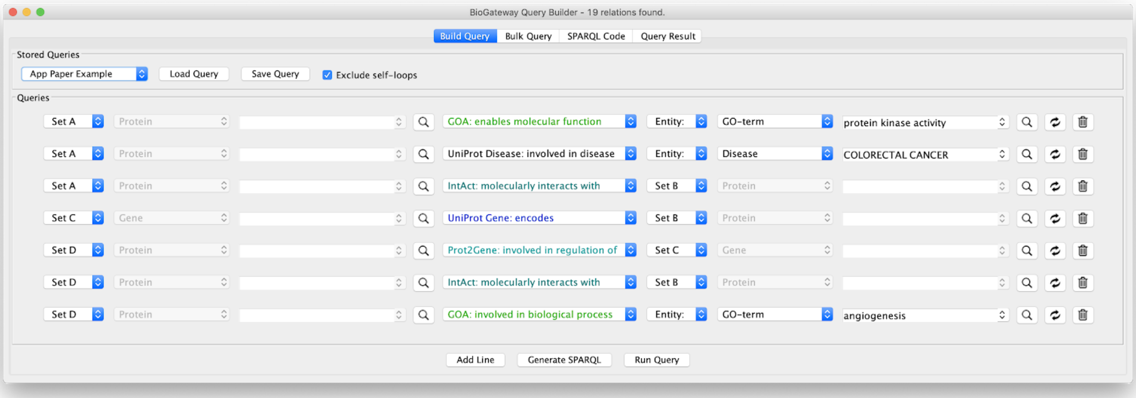

The final steps will be to click the button *Run Query*. This will open the *Query Results* tab, where all results have to be selected. Click on the *Import to new Network* button to generate the Network with the results.

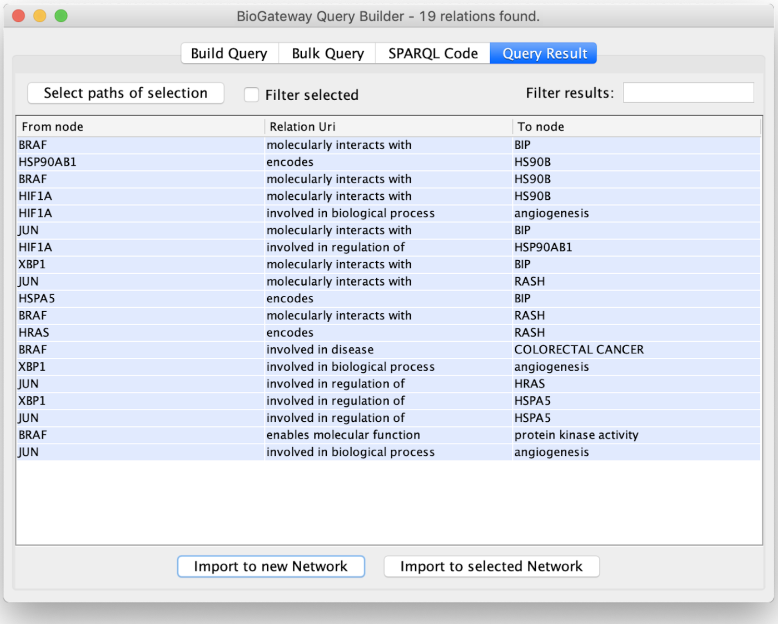

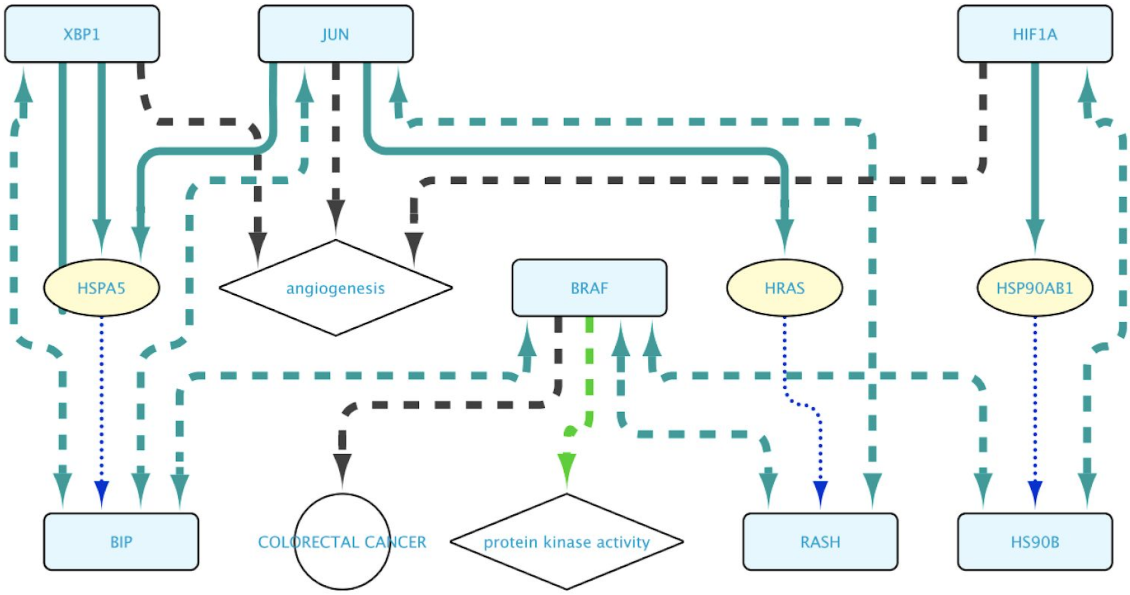

Edges representing relationships can be further explored by double-clicking on them. This allows to check the data supporting them, among other options. In this case, double click on the edge connecting XBP1 and HSPA5. This will create a new node representing the interaction. Right click-on it and select B*iogateway* > *Open resource URI*. This will open your web browser, showing the abstracts of the different papers supporting that interaction.

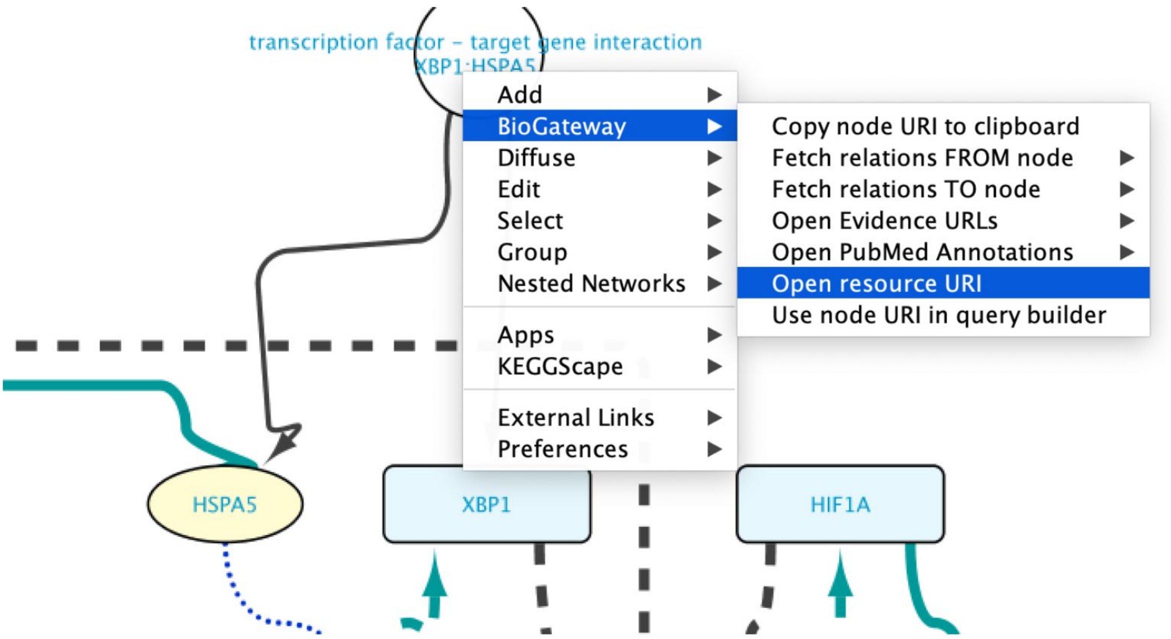

TRRUST

PMID: 12713871
Europe PMC Annotations

Activation of the ATF6, XBP1 and grp78 genes in human hepatocellular carcinoma: a possible involvement of the ER stress pathway in hepatocarcinogenesis.

BACKGROUND/AIMS

We identified the glucose-regulated protein (grp) 78 as a transformation-associated gene in hepatocellular carcinoma (HCC). Grp78 is a molecular chaperone involved in the unfolded protein response, the expression of which can be regulated by the transcription factors ATF6 and XBP1. Thus, we investigated the regulatory mechanisms of the grp78 gene in liver malignancy.

METHODS

Expression of grp78, ATF6 and XBP1 was examined by Northern blot, RT-PCR, immunoblot and immunohistochemical analyses. A reporter assay of the grp78 promoter was also performed.

RESULTS

Elevation of grp78 and ATF6 mRNAs and the splicing of XBP1 mRNA, resulting in the activation of XBP1 product, occurred in HCC tissues with increased histological grading. Higher accumulation of the grp78 product in the cytoplasm, concomitantly with marked nuclear localization of the activated ATF6 product (p50ATF6), was observed in moderately to poorly differentiated HCC tissues. Cooperation between the distal DNA segment and the proximal endoplasmic reticulum stress response elements was essential for maximum transcription of the grp78 promoter in HCC cells.

CONCLUSIONS

The endoplasmic reticulum stress pathway mediated by ATF6 and by IRE1-XBP1 systems seems essential for the transformation-associated expression of the grp78 gene in HCCs.

EXTRI

PMID: 18528784
Europe PMC Annotations
Highest score: 3.43187

Endoplasmic reticulum stress in the absence of calnexin.

Calnexin is a type I integral endoplasmic reticulum (ER) membrane chaperone involved in folding of newly synthesized (glycol)proteins. In this study, we used beta-galactosidase reporter gene knock-in and reverse transcriptase polymerase chain reaction (RT-PCR) to investigate activation of the calnexin gene during embryonic development. We showed that the calnexin gene was activated in neuronal tissue at the early stages of embryonic development but remained low in the heart, intestine, and smooth muscle. At early stages of embryonic development, large quantities of calnexin messenger RNA (mRNA) were also found in neuronal tissue and liver. There was no detectable calnexin mRNA in the heart, lung, and intestine. The absence of calnexin had no significant effect on ER stress response (unfolded protein response, UPR) at the tissue level as tested by IRE1-dependent splicing of Xbp1 mRNA. In contrast, non-stimulated calnexin-deficient cells showed increased activation of IRE1, as measured by RT-PCR and luciferase reporter gene analysis of splicing of Xbp1 mRNA and activation of the BiP promoter. This indicates that cnx (-/-) cells have increased constitutively active UPR. Importantly, cnx (-/-) cells have significantly increased proteasomal activity, which may play a role in the adaptive mechanisms addressing the acute ER stress observed in the absence of calnexin.

Example Use Cases

This section describes a diverse set of examples that are intended to illustrate the various functionalities of the Cytoscape Biogateway App and how they can be applied to answer biological questions through a network-based approach. Each of the use cases illustrates an example based on a biological question, translates it into a series of steps to a query and ends with the resulting network. To highlight the added value of the BioGateway App, we also briefly describe an alternative method that should result in (more or less) the same network. A compressed Cytoscape .cys file is provided in the following link to offer the possibility of further exploring the result of each use case. We also provide here a zip file containing all the query files for each of the use cases, so that they can be imported and tweaked for further use.
The App works best in the latest version of Cytoscape (3.7.0 or later). After downloading and installing Cytoscape from www.cytoscape.org you should install the BioGateway App as explained in the Installation page.

Use Case 1 - Exploring Around a Node

What is the biological context around the protein GTPase KRas encoded by the KRAS gene?

Starting from an empty network, the first step will be to right-click on the canvas and select BioGateway > Add a BioGateway node. Next, right-click on the canvas and select Biogateway > Add Biogateway node. The next step will be to enter the protein name RASK (all Capitals) in the query box (top of the BioGateway Node Lookup box, click Search, select the RASK protein and import by clicking Use Selected Node.

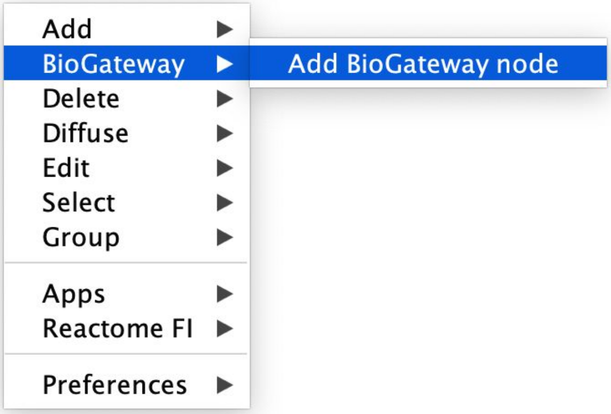

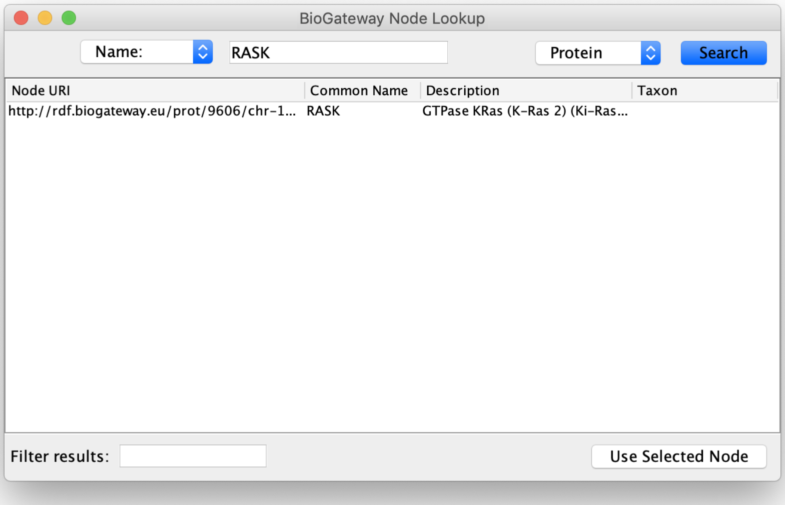

Once completed, right-click on the node in the Cytoscape network panel and expand, choosing from several options. In this step we select protein interaction partners from IntAct (right-click RASK, select 'Biogateway' > Fetch relations FROM node > IntAct: molecularly interacts with). Select all results and press Import Selected to display them to a network, adjust the display when necessary (e.g. Prefuse Force Directed Layout). The resulting network is shown below.

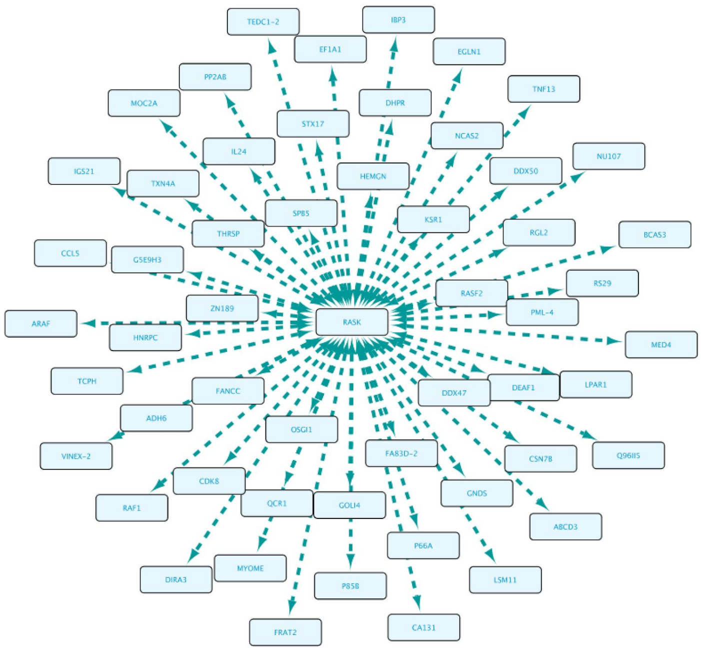

Now we will get the gene encoding for RASK and the TFs regulating it. To do so, right click on RASK and select Biogateway > Fetch relations TO node > UniProt Gene: encodes. Import the result, which then appears in the graph as a yellow oval indicating the gene KRAS. Now, right click on the KRAS gene and select Biogateway > Fetch relations TO node > Prot2Gene: involved in regulation of. Next, import all the results.

The last step will be to update the display (Prefuse Force Directed Layout) and add PPI interactions between all nodes in our network. This is done by selecting all nodes, right-clicking and selecting Biogateway > Fetch relations FROM selected > intact: molecularly interacts with. Finally, select all results, click Import relations between existing nodes.

The final network can be seen below.

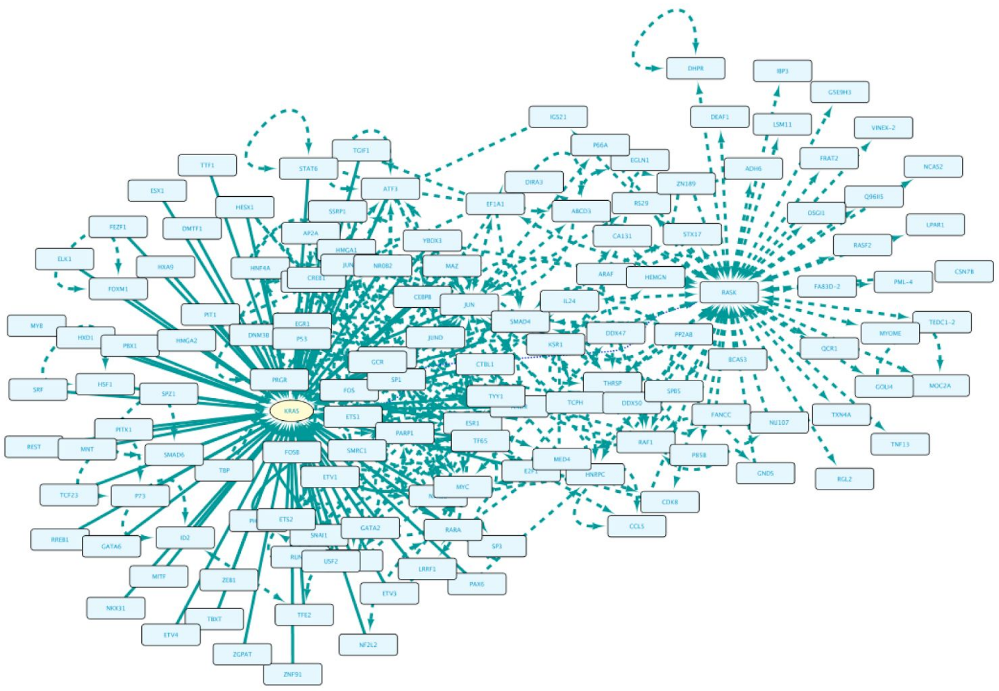

Non-BioGateway method:

1. Get interactions of RASK from Intact. Alternatively, get a previously built network containing RASK from repositories such as Reactome or others.
2. Find all TFs in the network. This can be done by finding GO annotations that relate proteins to a TF function.
3. Get the TGs of the TFs: use repositories such as TRRUST or TFactS to get the TGs from the TFs in our network.
4. Find PPIs between the TFs found in step 2 and the proteins in our network using IntAct.

Use Case 2 - Building a TF-TG network starting from a TF of interest

What genes are regulated by NFKB1? What is the literature supporting the results?

Create Query in Case2Query file (note that in the example we will only use a subset of the results). This query consists of one single line, where we ask for all the genes (Set A) whose transcription is regulated by NFKB1. Once we have defined the query, we click on the Run Query button.

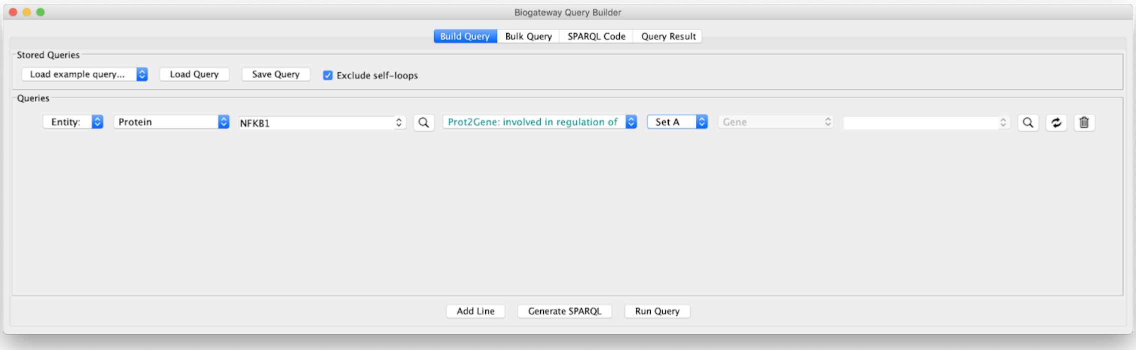

This will bring up the Query Result tab, where we select a subset of the target genes (HSPA1A, DCLK1, HTRA1, NOTO, HSD1182, MSTIR, CYP1A1 and WNT1). Next, we import the selected results by clicking on Import to selected Network, creating the network shown on the right.

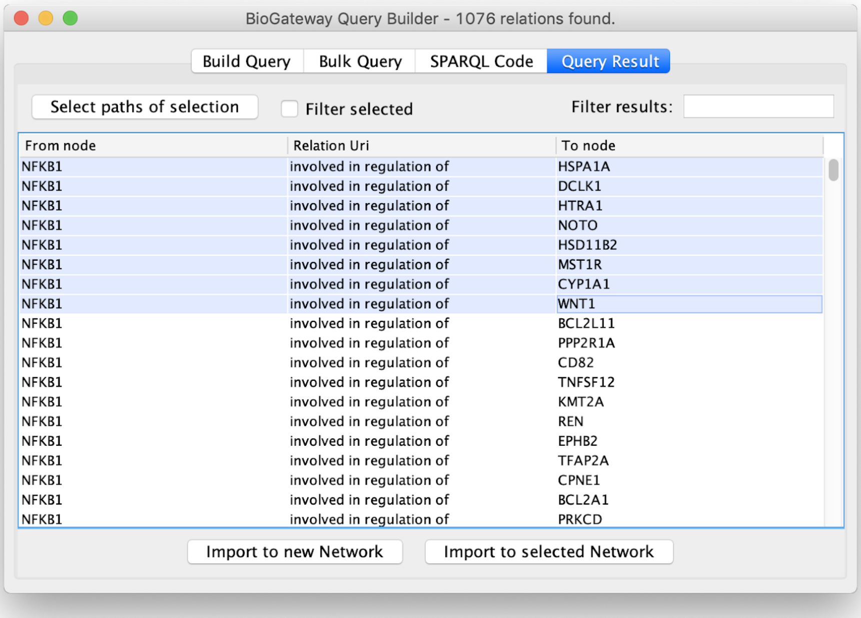

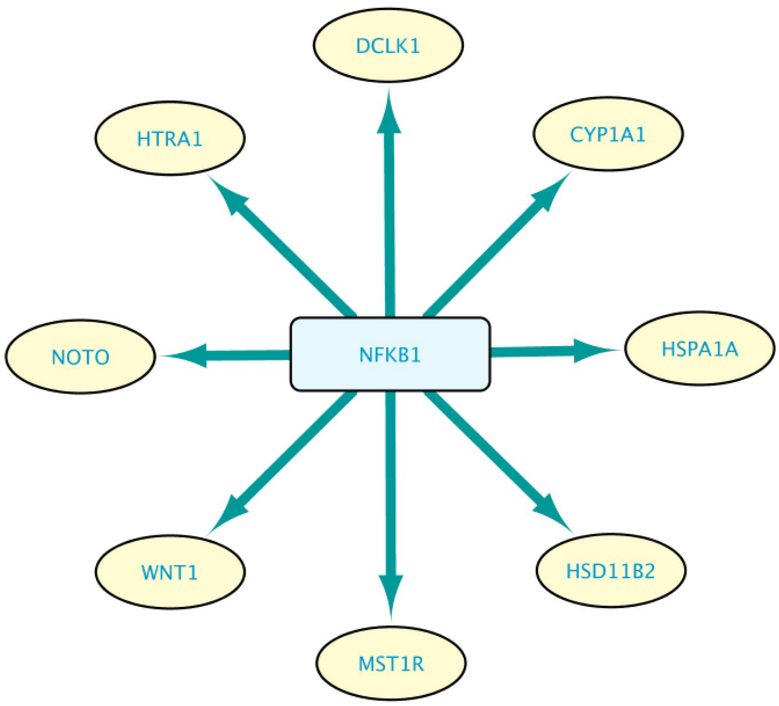

We can expand an interaction by double clicking on it. This will let us see more information supporting that interaction statement. The figure below shows the network after expanding the interaction between NFKB1 and WNT1.

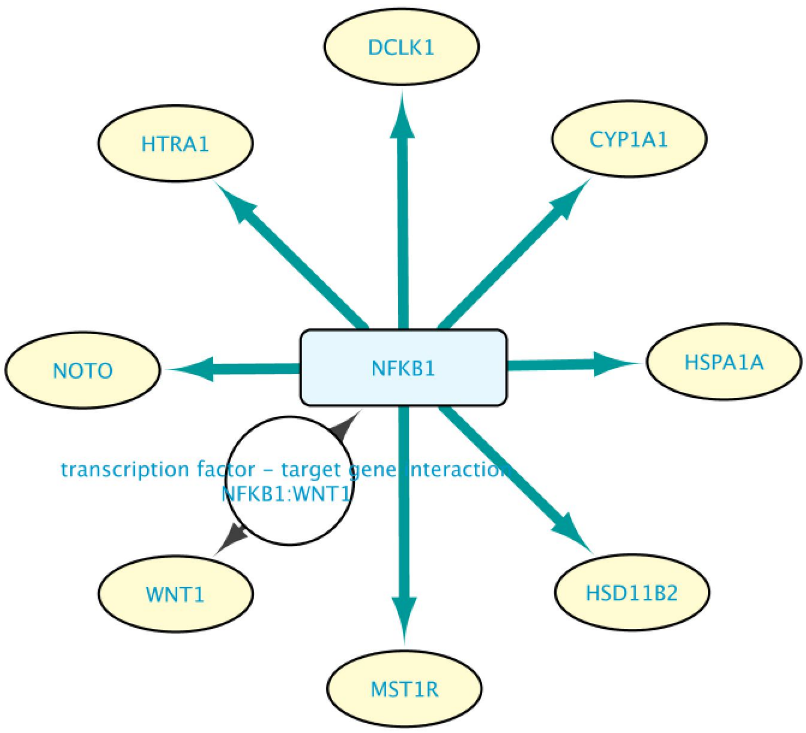

Right-clicking on the new node and following Biogateway > Open resource URI opens a tab in the web browser showing more data about that specific interaction. The top panel shows the identity of the TF and TG, normalised to standard Uniprot and HGNC names, with a

relationship provided by SIO (Semanticscience integrated Ontology, https://bioportal.bioontology.org/ontologies/SIO). The bottom panel shows the abstract, with highlighted in purple the sentence that was found by text mining (EXTRI resource, www.extri.org). The top-left of the bottom panel shows links to PubMed and the Europe PMC annotation platform.

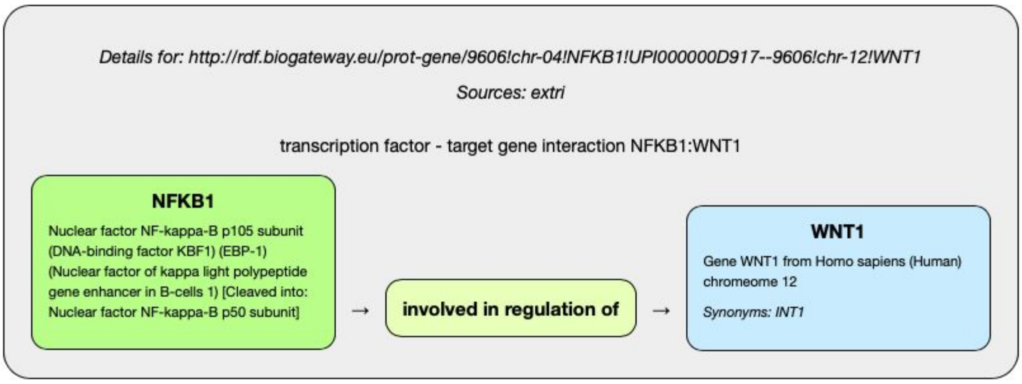

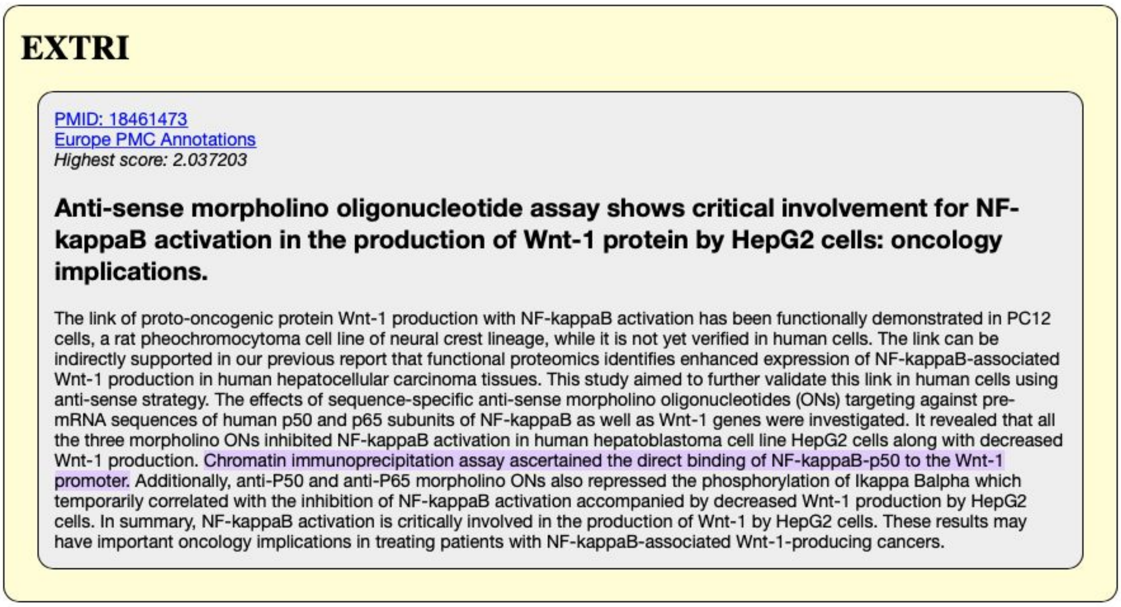

Non-BioGateway method:

1. Get TGs of a TF from a repository such as TRRUST.
2. Check the publications supporting a TFTG statement.

Further checking of the supporting evidence:

Whereas the Landing page provides an initial documentation of the evidence that supports why the TF-TG relationship was included in the BioGateway Prot2Gene graph, a user may link out to the PubMed abstract (PMID ID) or to Europe PMC, which will (soon) provide access to the annotated abstract through their SciLite curation platform (see figure below).

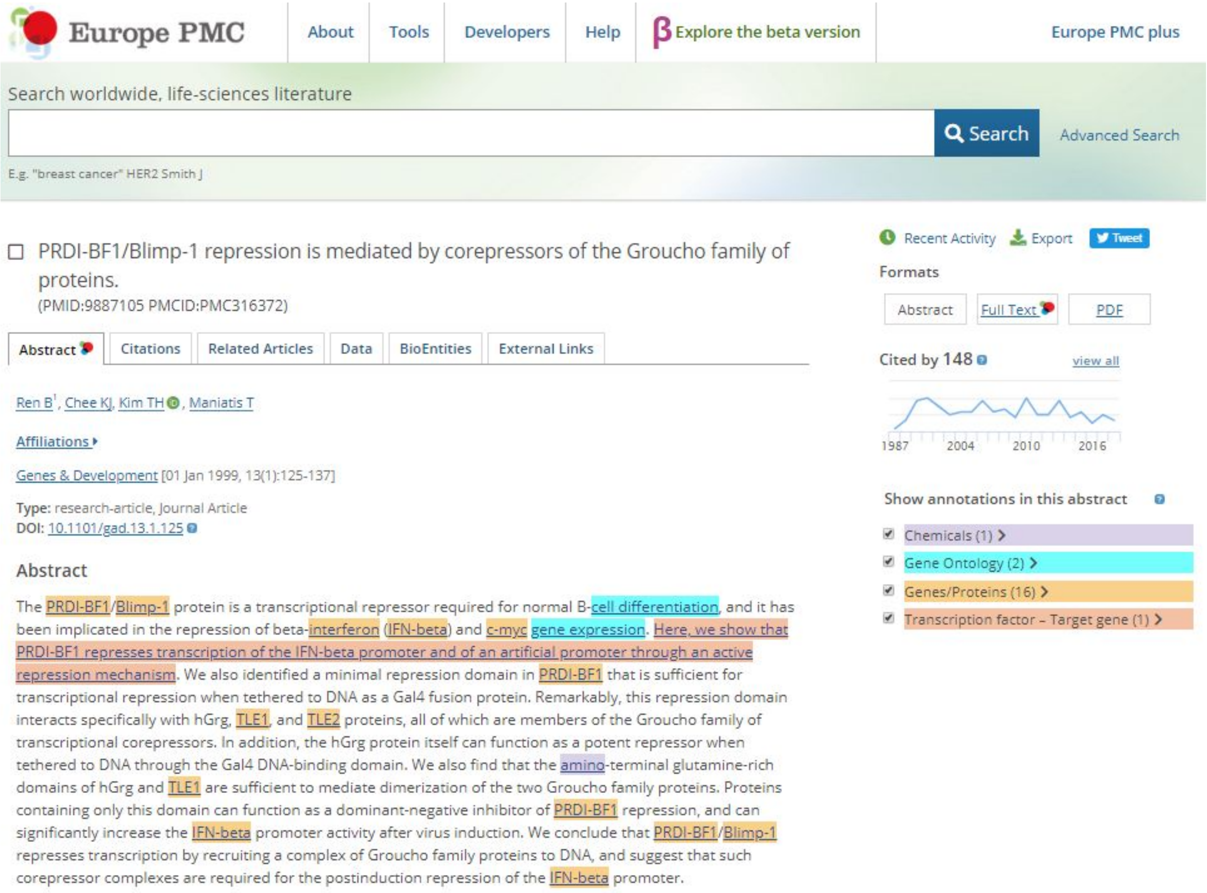

Europe PMC is developing the annotation platform SciLite. In the arbitrary example figure above, SciLite annotations are shown as highlighted pre-markings of chemicals, Gene Ontology terms, Genes and proteins, and a sentence that is part of the EXTRI resource (beta-version). The EXTRI effort (www.extri.org) will soon produce a new, high quality resource that we will upload in BioGateway. The sentence information will also be uploaded to the SciLite platform, where a user may indicate whether the sentence represents a bone-fide TF-TG relationship. The results of such a 'community-curation' effort will be incorporated via API back into BioGateway, where the confidence level (0, medium or high) will be updated.

Use case 3 - Establishing Molecular Relationships between Diseases

What proteins are involved in both Breast cancer and Colorectal cancer? What TFs regulate the transcription of the genes of these proteins?

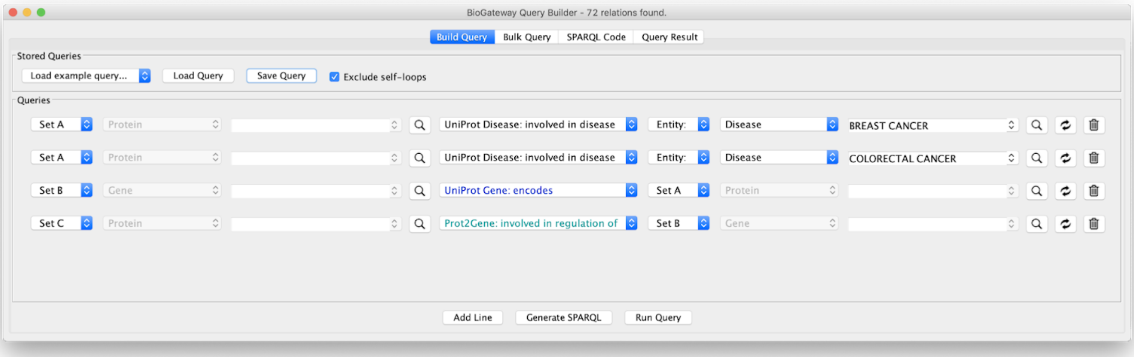

Create the query below or import from file Case3Query, which will get all proteins known to be involved in both Breast cancer and Colorectal cancer, the genes encoding for those proteins and the Transcription Factors regulating these genes. The resulting network can be seen below.

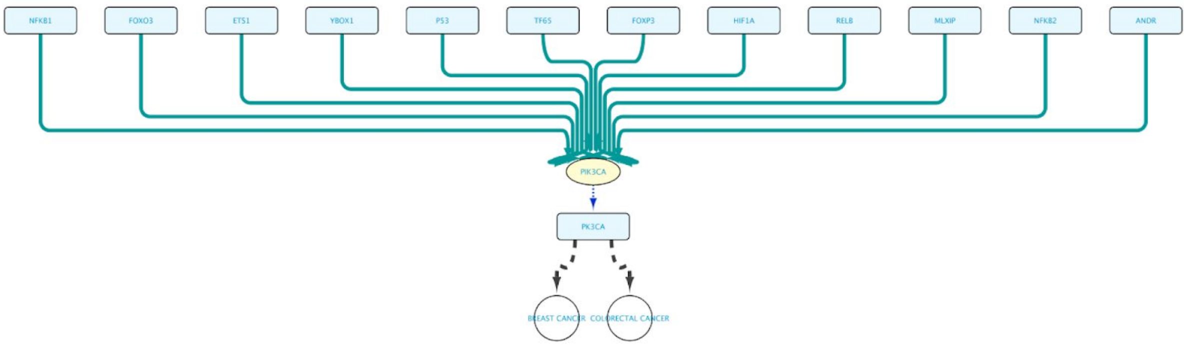

Non-BioGateway method:

1. Get proteins annotated to be involved in the desired diseases in a protein to disease repository.
2. Find common proteins in both networks.
3. Get the genes encoding for the found proteins in repositories like Entrez Gene.
4. Get the TFs regulating these genes in repositories such as TRRUST or TFactS.

Use case 4 - Find the Proteins connecting two GO Terms

Can we find the proteins that form a bridge between two distinct biological processes (cell adhesion to extracellular matrix and G1/S transition)?

The first step will be to create the query below, or load the query in file Case4Query.

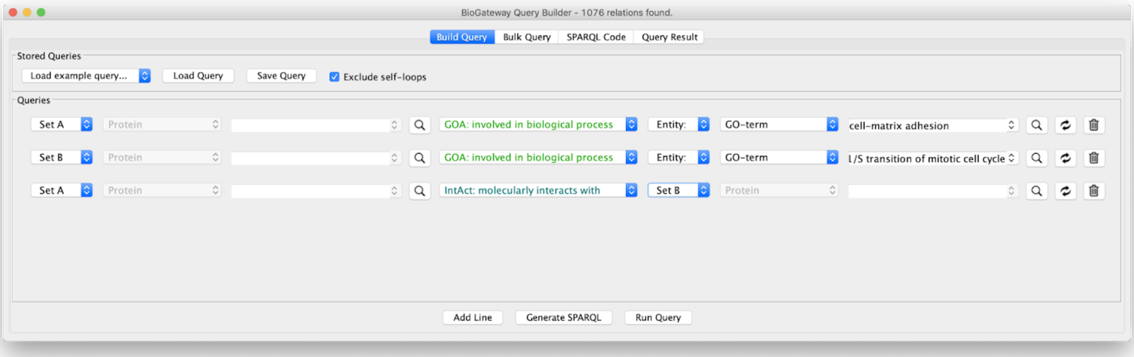

Running the query and importing all results will produce the following network:

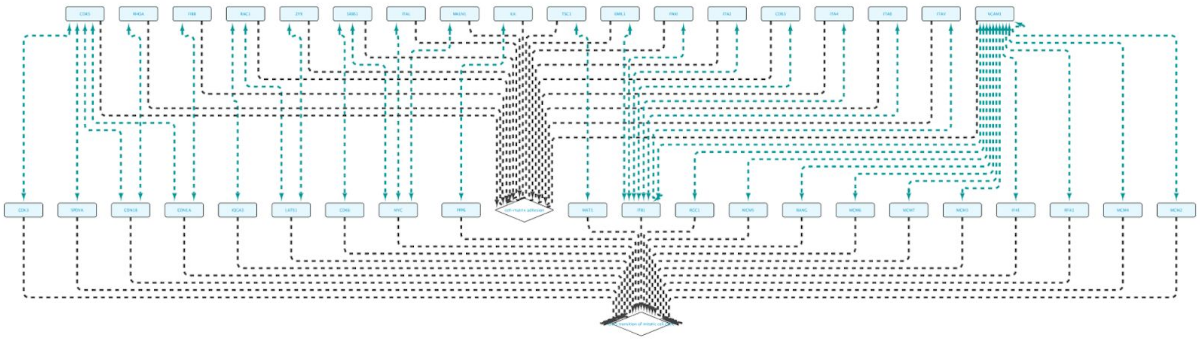

Non-BioGateway method:

1. Find proteins annotated to the two GO terms separately.
2. Look for common proteins to get the connections.

Use case 5 - Find proteins with protein kinase activity involved in a disease and the context around them

What proteins with a kinase function are involved in colorectal cancer? What is the biological context around these proteins?

Build the query below or load the Case5Query from file, run the query and import all results.

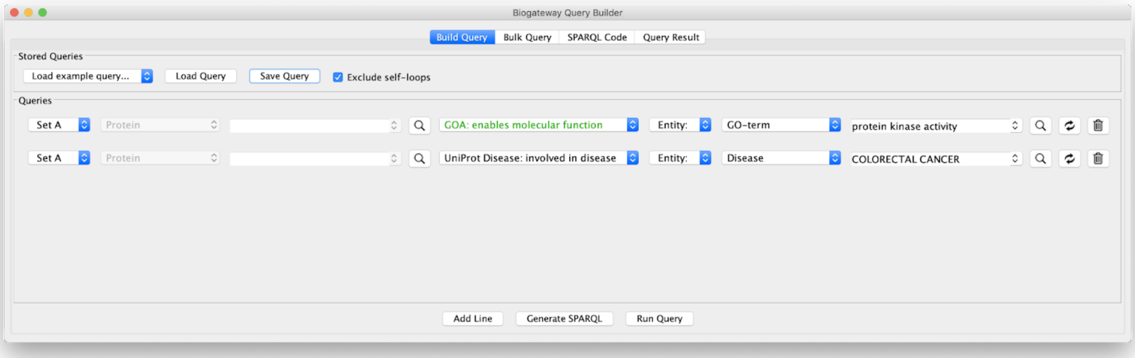

The result of this query can be seen in the image below: the protein BRAF, which has a protein kinase function and has an involvement in colorectal cancer.

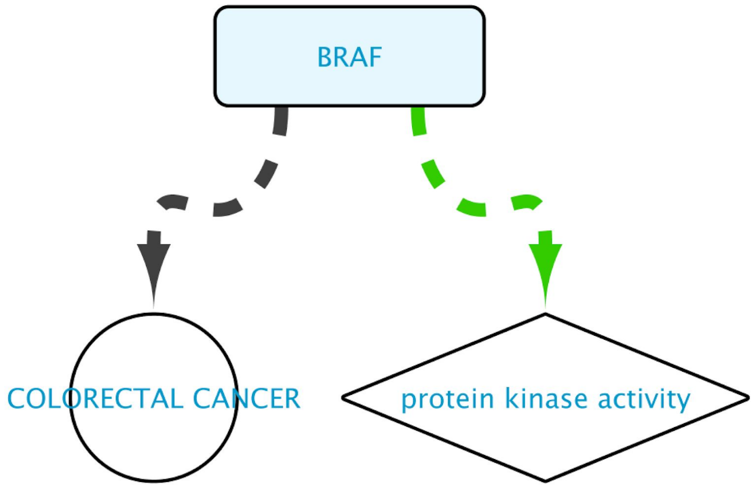

Now we will extend this small network by following a series of steps. The first one will be to get PPIs involving BRAF. Select this node and right-click Biogateway > Fetch relations FROM selected > IntAct: molecularly interacts with. Import all results. Next we will get the Target Genes of the Transcription Factors in the network. Select all nodes and Biogateway > Fetch relations FROM selected > Prot2Gene: involved in regulation of. Import all results.

The next step will be to get the proteins encoded by the regulated Target Genes and look for interactions with the existing proteins in the network. To do so, select all nodes and Biogateway > Fetch relations FROM selected > UniProt Gene: encodes. Import all results. Next, select all nodes again and Biogateway > Fetch relations FROM selected > IntAct: molecularly interacts with. Import relations between existing nodes.

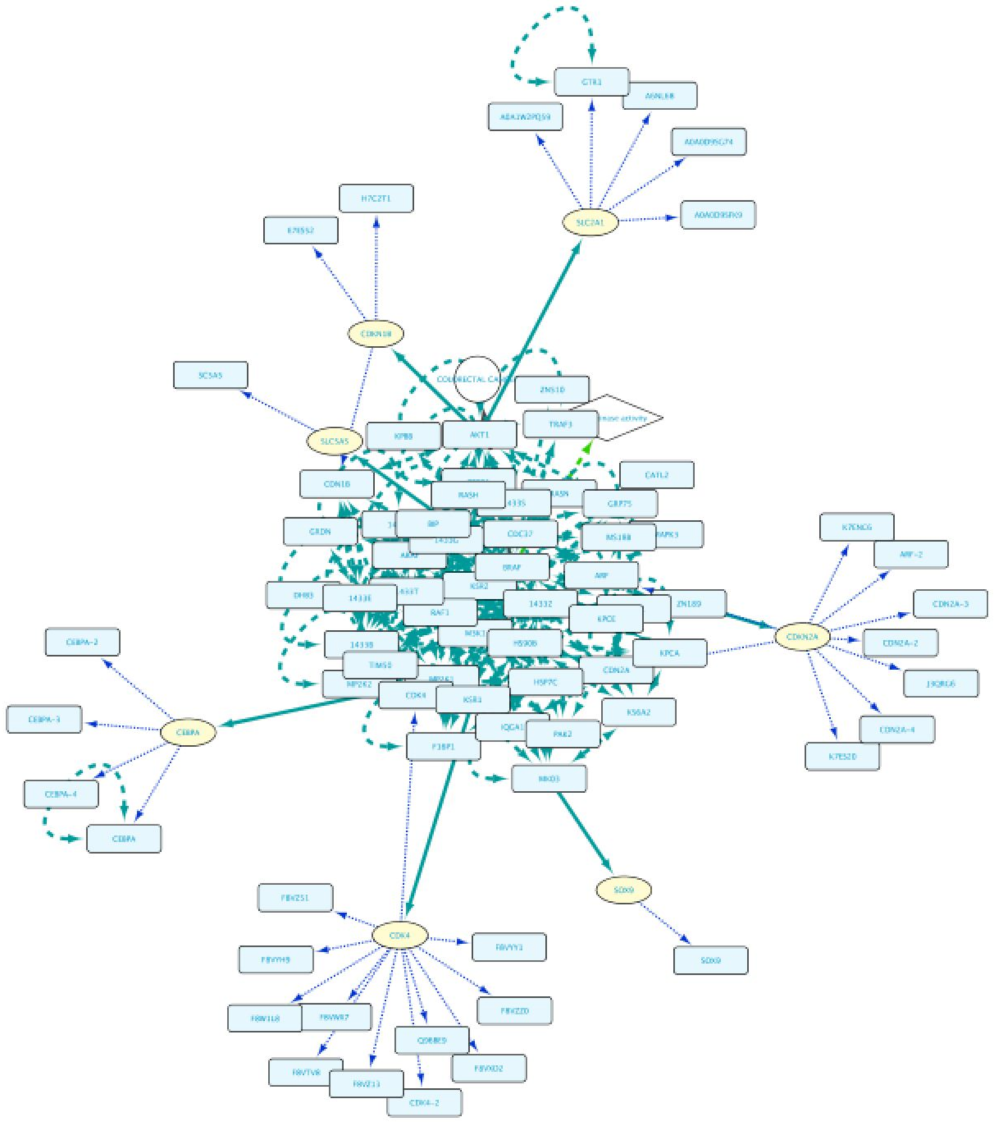

Non-BioGateway method:

1. Find proteins annotated to the GOA protein kinase activity.
2. Get proteins involved in diabetes-mellitus, non-insulin-dependent.
3. Find common proteins in both networks.
4. Find TGs of IRF7 in repositories like TRRUST, TFactS or others.
5. Get proteins encoded by these TGs in Entrez Gene.
6. Look for all PPIs involving the nodes in the network and find the ones involving the proteins in the built network.

Use case 5 (Extension) - Generalise a network from BioGateway for further analysis with Cytoscape apps

What potential protein complexes can I find using MCODE on a network created by Biogateway?

We will extend the network from the use case 5. Thus, the first step will be to load, run and import all the results from the Case6Query. The next step will be to find all the genes regulated by the Transcription Factors in our network. Select all the nodes, right click and Biogateway > Fetch relations FROM selected > Prot2Gene: involved in regulation of. Import all relations.
Next, we will find the genes encoded by the genes in the network. Select all the nodes, right click and Biogateway > Fetch relations FROM selected > UniProt Gene: encodes. Import all relations. The following will be to find protein interactions between the proteins in our network. Select all the nodes, right click and Biogateway > Fetch relations FROM selected > IntAct: molecularly interacts with. Import connections between existing nodes.
Finally we will use the MCODE cytoscape plugin, which uses network analysis to look for potential complexes in a network. We will leave the default settings except that we will enable the option Include Loops. Next we will click on Analyze Current Network and create a sub-network with the first result.

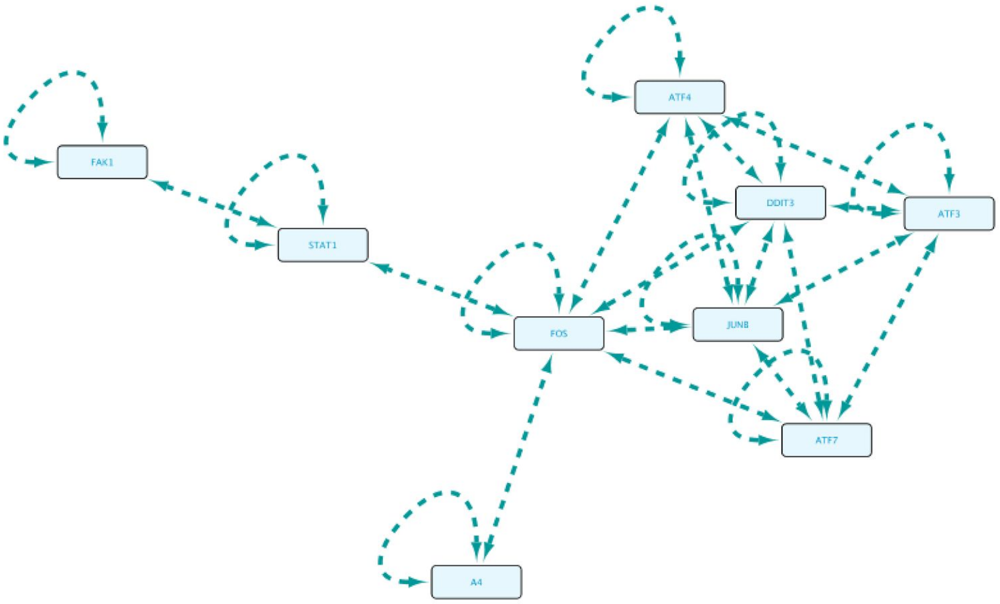

Use case 6 - Connect Protein A to Protein B (with GO restriction C)

What PPIs can we find between proteins annotated to be involved in the G-protein coupled receptor signaling pathway?

Create the query as stored in file Case6Query, which finds all proteins involved in a specific GO term. The example shows the GO term “G-protein coupled receptor signaling pathway”.

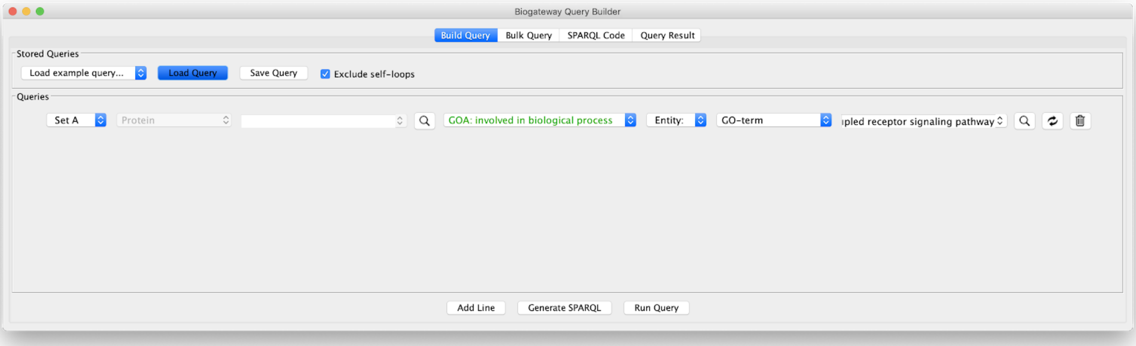

Now, we will look for PPIs between the nodes in the network: select all nodes and Biogateway > Fetch relations FROM selected > IntAct: molecularly interacts with. Import relations between existing nodes. Finally, we will create a subnetwork containing only the nodes connected by PPIs by using the Cytoscape filtering tool (Cytoscape Control panel > Select, see image below). This will create the subnetworks seen in the image on the right.

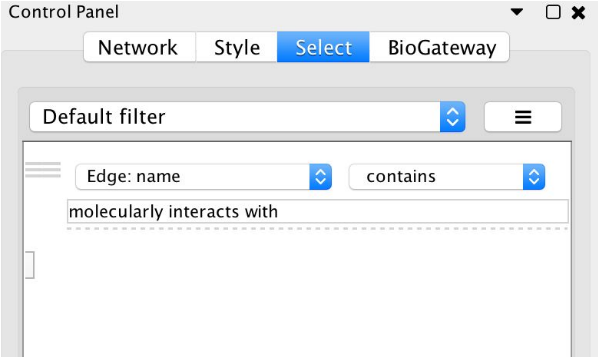

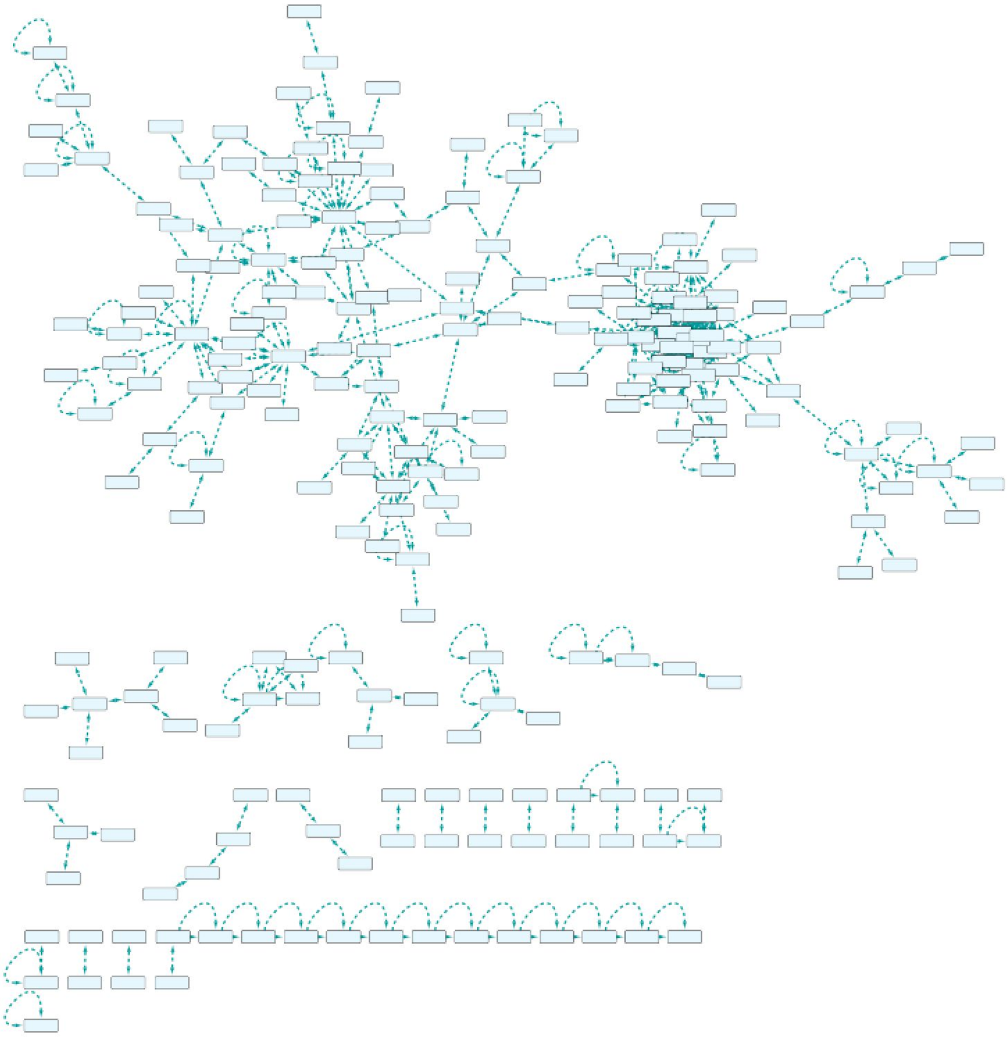

Non-BioGateway method:

1. Find all proteins involved in the GO term of interest.
2. Find connections between them on IntAct.

Use case 7 - Potential downstream effects after targeted inhibition of proteins

What are the downstream effects of inhibiting MAP3K7 and AKT1? Can we find a connection between the two affected proteins?

Create the query below, as stored in the file Case7Query. You will notice that we have not specified a specific biological process term in line 5 of the query, which allows any annotation term in the results. Next, we can retrieve a subnetwork from the results by filtering for apoptotic process.

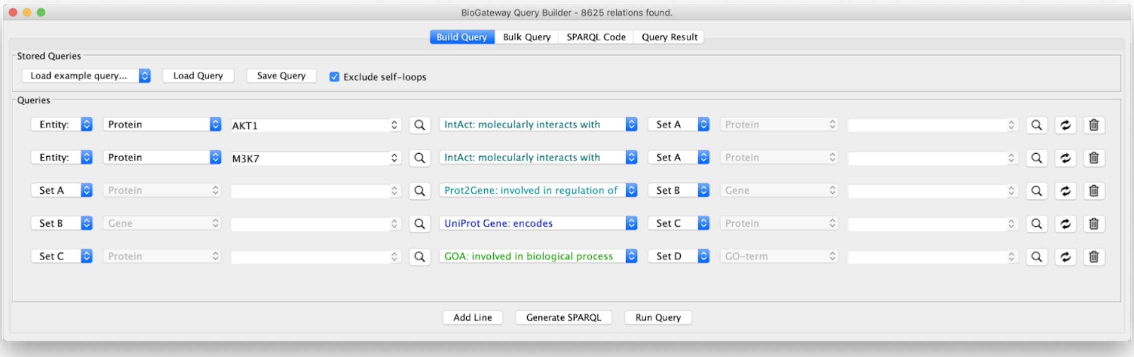

In the Query Results tab, we will use the Filter results text field, where writing DNA damage will select all GO biological process terms containing DNA damage.

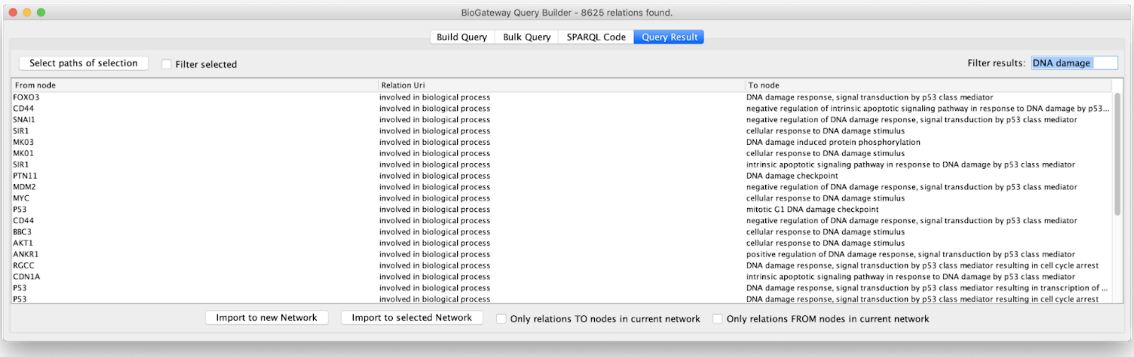

Now we will select all the relations in the results table and click on the Select paths of selection button on the top left corner. This will select only the relations creating the subnetwork we are interested in. Next, we click on Import to new Network. The resulting network can be seen on the right.

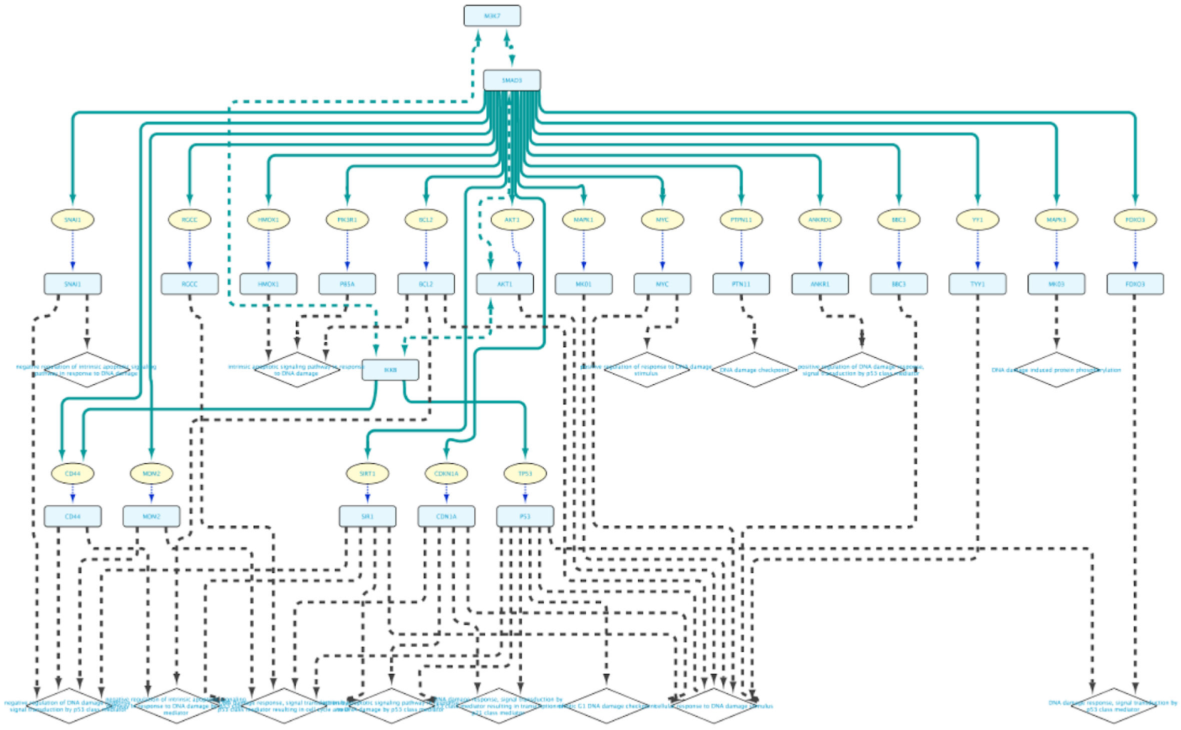

Non-BioGateway method:

1. Check repositories of large-scale transcriptome data for transcriptome profiles measured after exposing cells to both drugs and perform functional enrichment analysis to reveal the affected biological process.
2. Identify the targets in a protein-protein interaction (e.g., IntAct), identify which interacting partners are TFs (TFclass or GO [GO:0000981]), find the TGs (HTRIdb, TRRUST etc) and then, finally, check for the GO BP terms associated with the TGs (Uniprot, RefSeq, GO, GeneCards etc).

Application Manual

Create your own query

So far we have been working with pre-built queries to understand the way they work. This section will show how to build a query from scratch to create a network around the genes, proteins, etc of interest for you.

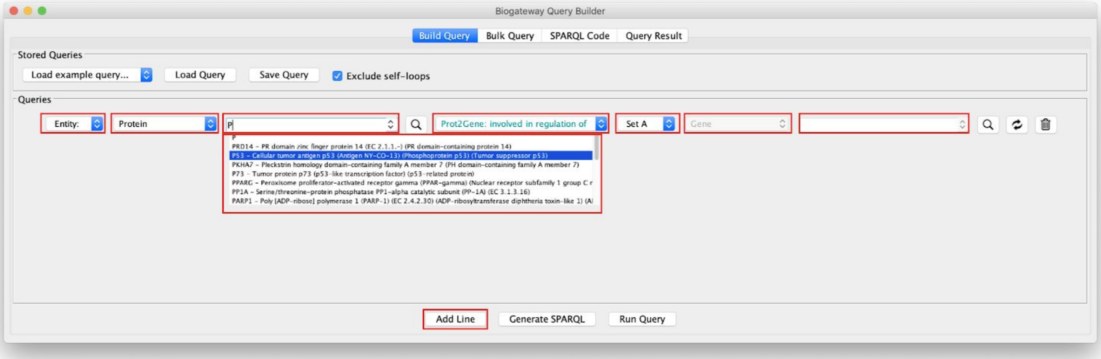

When building a query, it is mandatory to specify the following parameters:

- Entity or Set: this has to be set for both the Subject and the Object. This allows to select whether the Subject/Object is a specific biological entity (e.g. a specific protein, gene, etc) or a Set of entities. In the figure above the Subject is defined as Entity and the Object is defined as Set A. Note that the number of possible Sets shown in the drop-down menu will increase when more sets have been identified.
- Entity type: this value is set automatically depending on the relation type. Valid entity types will be displayed in black, while invalid entity types will be displayed in red. In the example shown in the figure above, the Subject is a Protein and the Object is a Gene because the selected relation type is Prot2Gene: involved in regulation of.
- Entity name: this text field will only be enabled when Entity is selected at the corresponding dropdown menu. In the example, only the Subject is set to Entity, which enables the field for Node lookup only for the Subject.
  In this field, users can write their protein, gene, etc of interest to find it in BioGateway. Typing in the field will trigger an autocomplete search engine that will aid in the selection of the node of interest. Once found, just click on the node of interest to select it.
- Relation type: this dropdown menu allows the user to select the type of relation between Subject and Object.

More lines can easily be added by clicking the Add Line button, whereas a line can be deleted by clicking on the trash bin icon on the right side of the line to delete. Furthermore, the Subject and Object can be swapped by clicking the swap button on the right side of the line to swap.

Creating Advanced Queries

The BioGateway App offers the possibility of specifying in more detail the Active Properties and additional Query Constraints to create more advanced queries. This is done through the BioGateway tab in the Control Panel of Cytoscape.

To be able to specify Query Constraints, the user will need to activate one or more of the Query Constraint branch nodes of the Active Properties tree. This will display the Query Constraints section in the lower panel of the Control Panel. The next step will be to check the constraints of interest and set the value for each selected constraint. To understand the meaning of these values and how each score was computed, the user should visit the documentation for each of the resources.

After setting all the desired values, they are active for the next query that is run: simply open the Query Builder window, import or build a query and run it. The results of the query will be filtered according to the set parameters.

It is worth noting that creating advanced queries can be a trial and error process that needs some tweaking until a satisfactory set of parameters is found.

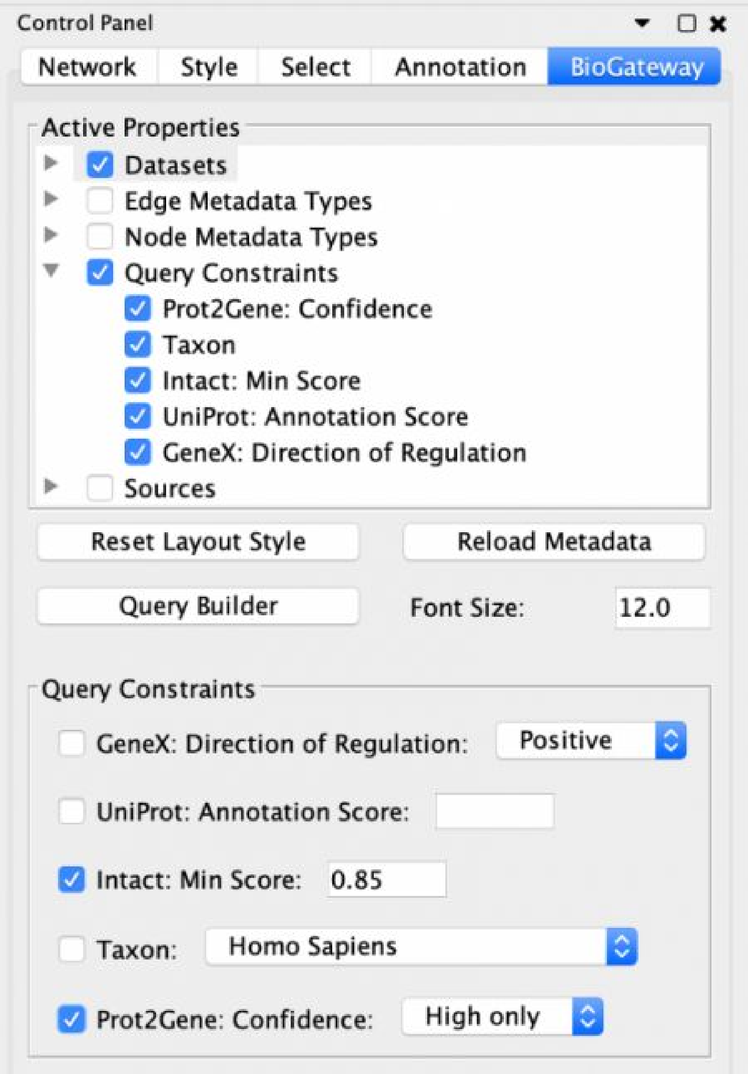

The Exclude self-loops Setting

When creating a query in the Query Builder, the option Exclude self-loops will be on by default, as this better reflects the logics behind the query building. In addition, we have also experienced that allowing self loops in some cases significantly increases the number of results, so the default setting safeguards against that.
The Exclude self-loops option forces the different Sets specified in the query to be disjunct, meaning not overlapping with each other (e.g. Set A and Set B will never contain an instance of the same entity). However, in Biology there are many cases where self-loops are important to consider, for instance, when a protein forms a homodimer to be able to perform its function. This specific case would be depicted as a self loop in BioGateway, and is not part of the results when the Exclude self-loops option is enabled.
We recommend that once a query yields interesting results, a comparison is done with a network generated while disabling the Exclude self-loops option.

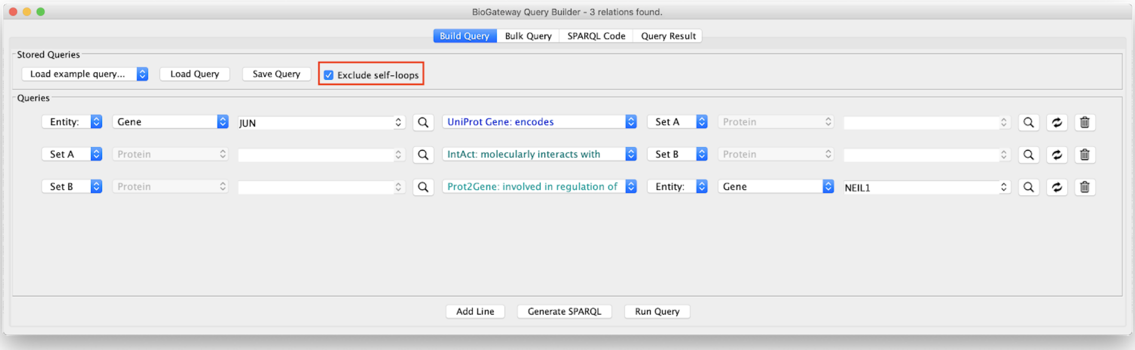

The image above displays an example query to showcase the function of this feature. Line 1 asks for the Protein encoded by the JUN Gene. Thus, Set A will only contain the Protein JUN. Line 2 asks for a Set B containing the Proteins interacting with Set A (JUN). Finally, line 3 restricts the Proteins in Set B to only those involved in the regulation of the NEIL1 Gene Expression.

Exclude Self-loops option enabled

In this case, the intersection between Sets A and B will be discarded from the results. This means that Set B can not contain JUN. The image below shows the resulting network.

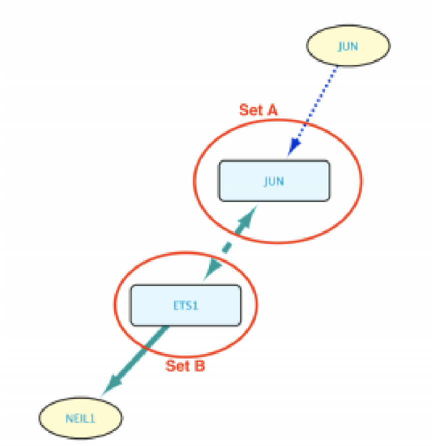

Exclude Self-loops option disabled

In this other case, the intersection between Sets A and B is allowed to be part of the results, meaning that Set B contains JUN, which is subsequently taken into account when searching for genes regulated by Set B members. The resulting network can be seen in the image below.

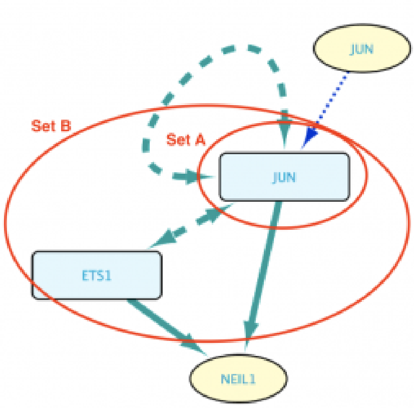

Saving/Loading queries

The Load example query box of the Stored Queries section of the Query Builder (below) contains several saved queries that can be loaded for demonstration and exploration purposes. The Load Query button allows a user to load previously saved queries, and the Save Query button allows saving a newly built query for future use.

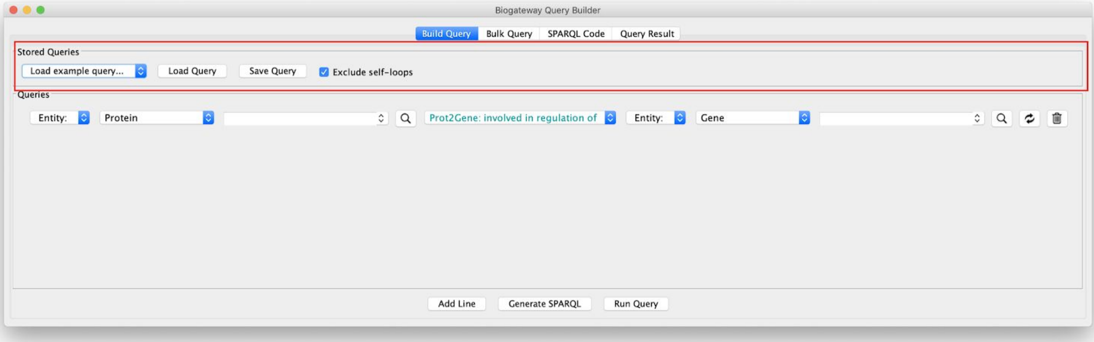

Saving queries

A query can be saved in a file once it has been built in the Query Builder. Click on the Save Query button. This will open a dialog that allows the user to select the directory where to store the query, as well as naming it as a .bgwsparql file. When having decided the name of the file and the directory where to save it, click the Save button to save the query.

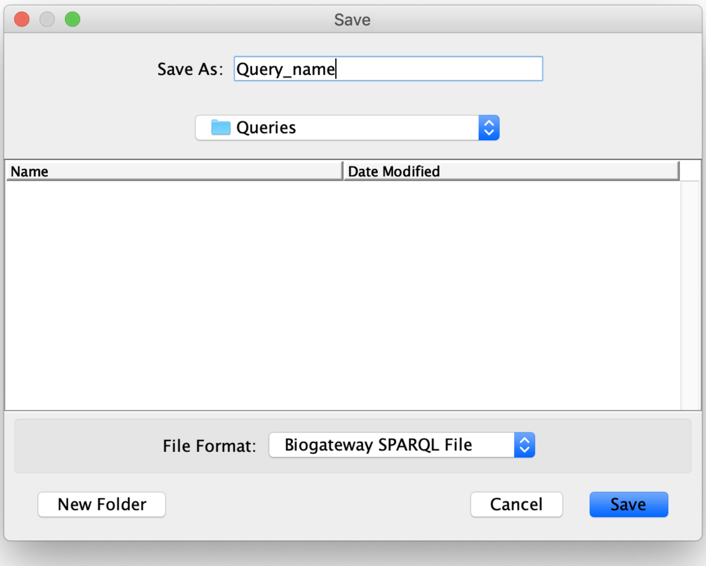

Loading queries

A previously saved query can be loaded by clicking the Load Query button. This will open a dialog that will allow the user to navigate to the desired directory and select the query of interest. After finding the query to load, select it and click on Open.

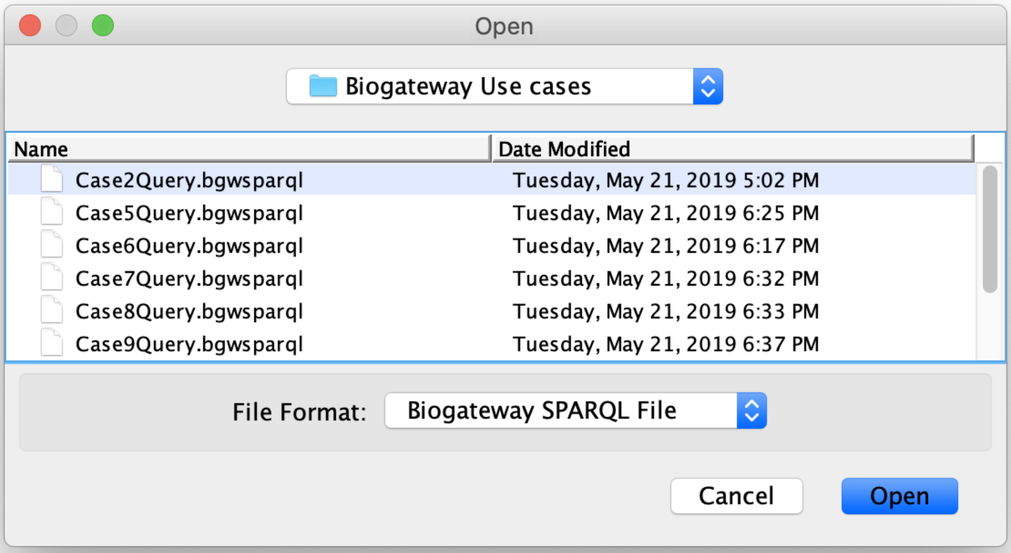
Open
Biogateway Use cases
Name
Date Modified
Case2Query.bgwsparql
Tuesday, May 21, 2019 5:02 PM
Case5Query.bgwsparql
Tuesday, May 21, 2019 6:25 PM
Case6Query.bgwsparql
Tuesday, May 21, 2019 6:17 PM
Case7Query.bgwsparql
Tuesday, May 21, 2019 6:32 PM
Case8Query.bgwsparql
Tuesday, May 21, 2019 6:33 PM
Case9Query.bgwsparql
Tuesday, May 21, 2019 6:37 PM
File Format:
Biogateway SPARQL File
Cancel
Open

The SPARQL behind the scene

In some cases the user might want to extract the SPARQL code for the query built in the Query Builder. This can easily be done by clicking the Generate SPARQL button right after having finished building the query.

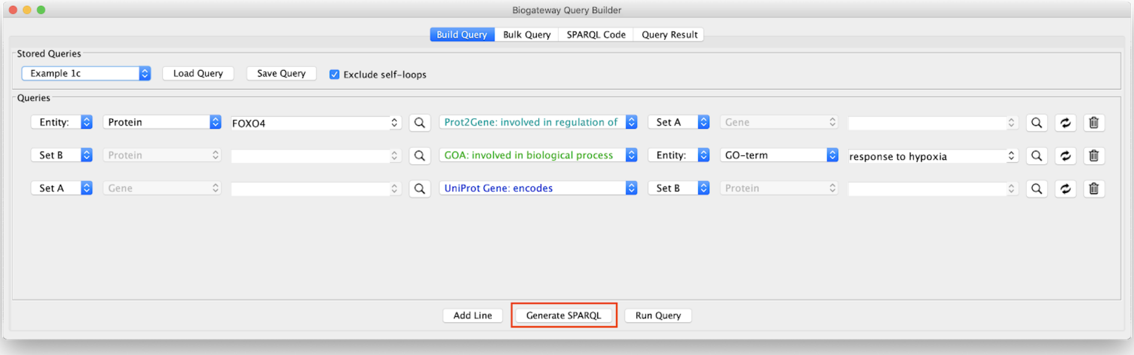

This will redirect the user to the SPARQL Code tab of the Query Builder window, where the complete code that has been generated is displayed.

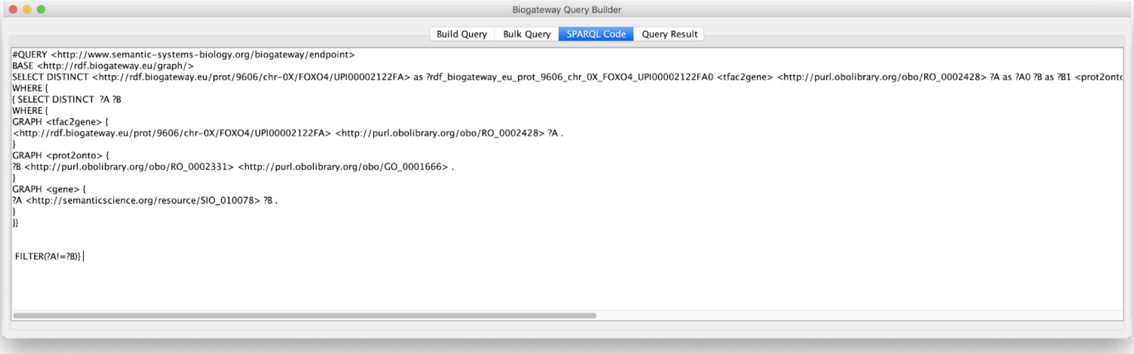

Query Builder Results

A query in the BioGateway Query Builder is launched to the server once the user clicks the Run Query button. Once all the results are found, the Query Builder window will show the Query Result tab, where the results are displayed and can be inspected prior to selection and importing them to a Network in Cytoscape.

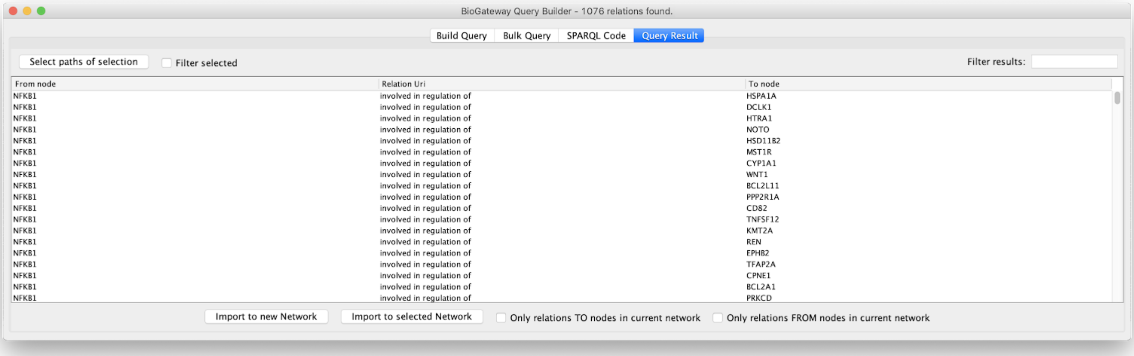

The Query Result tab offers the user the option to select all, or only a subset of the results to be imported as a network. Specific results can be selected by Ctrl/Cmd + click the rows of interest and/or Shift + click the rows of interest. Alternatively, all results can be selected simply by using Ctrl/Cmd + A. Next, the selected results can be imported to a network in Cytoscape by clicking the Import to new Network (to import the selected results to a new network in the Cytoscape session) or Import to selected Network buttons (to import the selected results to the currently active network in the Cytoscape session).
The selection of subsections of the results can be further facilitated by sorting on the different columns in the results. Also, the Filter selected checkbox will hide all unselected rows to ease reviewing the rows to import.

Filter results based on nodes in selected network

When using the Query Builder to find new nodes to add to an existing network, it will often be useful to restrict the results to be connected to some of the nodes already present in your selected network. The Query Builder Result panel includes two check-boxes for this purpose.

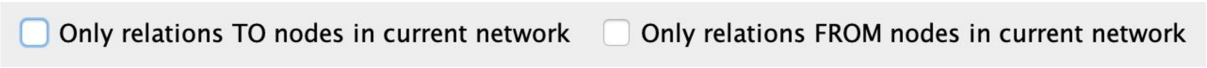

Selecting either check-box will limit the new results to relations with nodes already present in an active network.

Only relations TO nodes in current network

Checking this box will filter the result list to only show the relations where the target node (right-most column) is present in the currently selected network.

Only relations FROM nodes in current network

Checking this box will filter the result list to only show the relations where the source node (left-most column) is present in the currently selected network.

Text Filtering

Sometimes queries will produce a challenging number of results. A text filtering function is designed to ease the process of finding specific results in the Query Result tab. In the Filter results box located in the top right corner of the Query Result window, the user can write a text of interest to filter results by. Results that match the introduced text in any of the columns will be selected. This can significantly reduce the number of result lines displayed in the Query Result window, allowing a user to home in further to the results of interest.

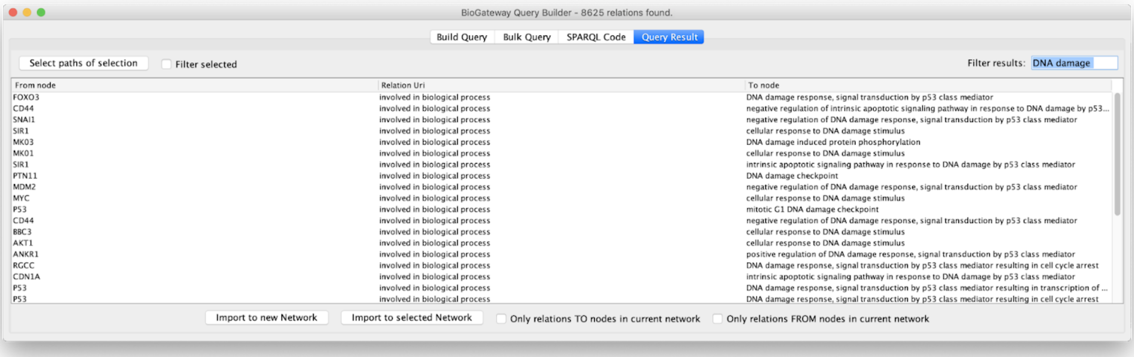

Select Paths

When a query retrieves a large number of results it can be useful to import only a subset of these. As explained previously, the user can select one or multiple rows by clicking on them. However, it can be useful to also include all the nodes and edges leading to the selected rows. The Select paths of selectionbutton allows the user to automatically find all nodes and edges in the results that mark a directionality to the rows that have been selected by the user.

To do so, first select the rows of interest in the Query Result tab.

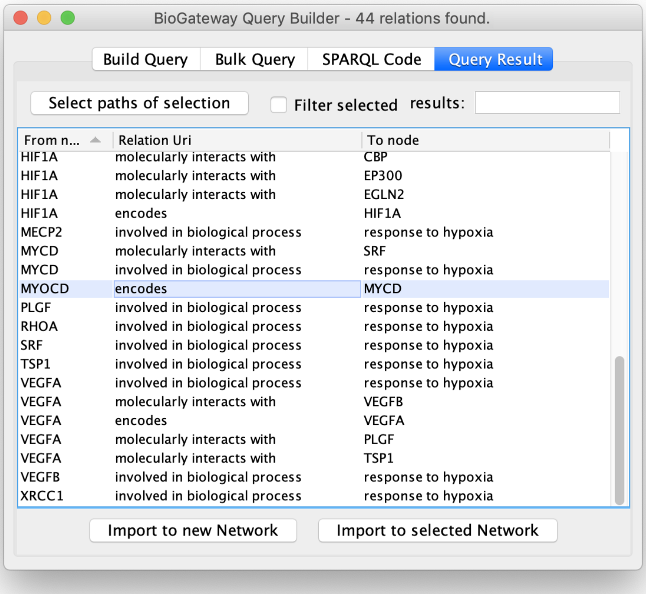

Next, click the Select paths of selection button to find all the results pointing to the selected rows.

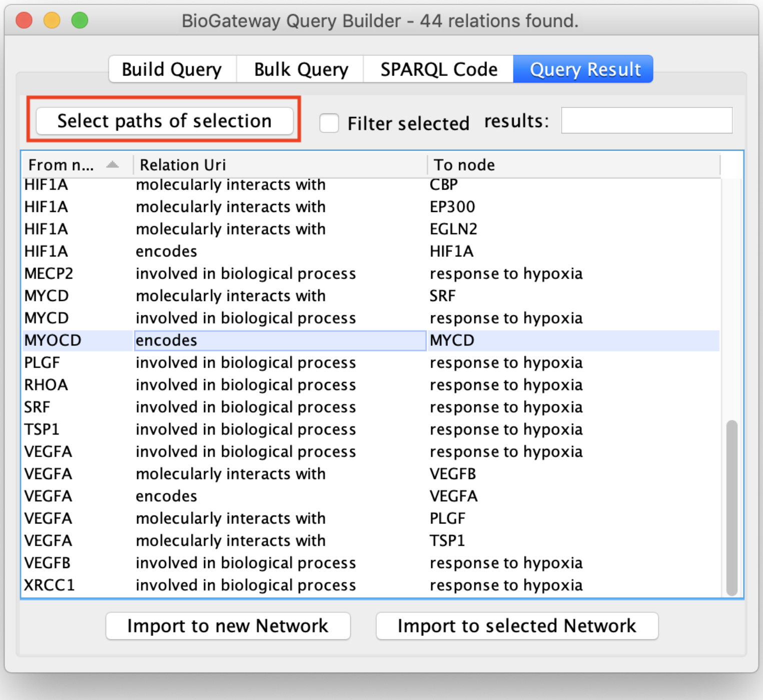

All the results will be displayed again, but only the rows of interest will be selected.

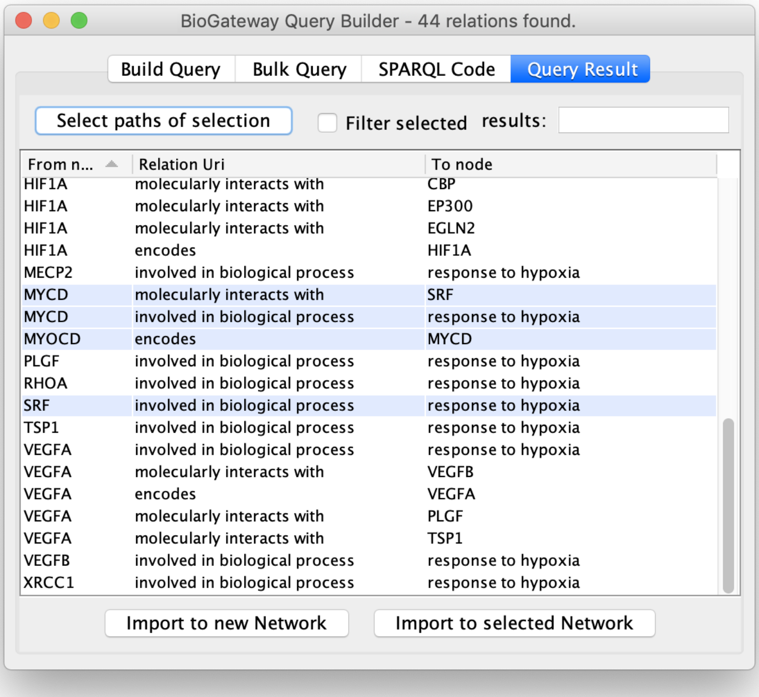

As a result, the user will get a network containing the nodes and edges leading to the row(s) initially selected in the Query Result tab of the Query Builder window.

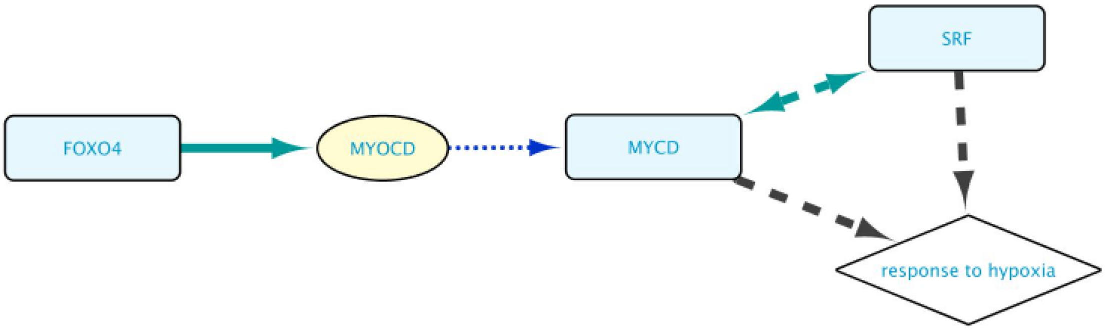

Reviewing relations

Some types of relations such as Protein-Protein interactions or Transcription Factor-Target Gene interactions are derived from database resources and publications. When building a network it is important to know which resources support a relation and what the publications are behind that information. The BioGateway App offers the possibility to easily retrieve the provenance of these relations, allowing the users to assess the source of the data and decide if they want to keep or remove a relation from their network based on their own criteria. All this is done via a double click expansion of edges in a BioGateway network.
When interested in the background of the data supporting a specific relation in the Imported Network, the user will only need to double-click on the edge representing that relation. This will expand the edge, creating a new node that will represent that interaction. The new node can now be accessed by right clicking it, which brings up several options in a drop-down menu.

Going to the source of the data

The original source of the data can be easily accessed by clicking on a node and selecting Biogateway > Open Evidence URL. This will open a Web Browser window leading the user to the resource supporting that interaction, allowing further exploration of the methods used to detect the interaction, etc.

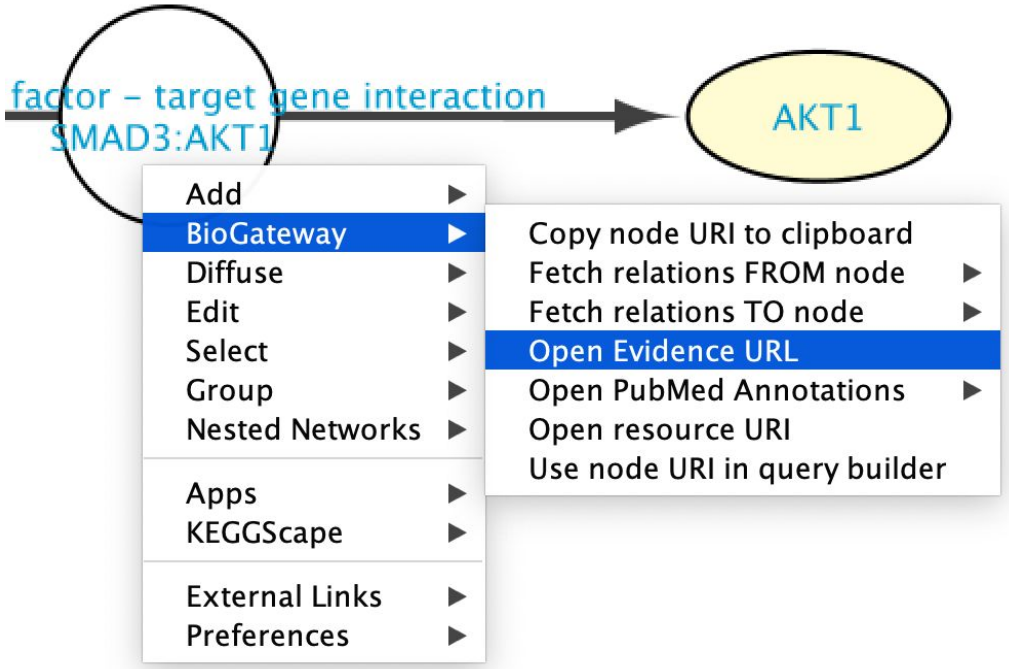

Going to PubMed articles

PubMed articles supporting an interaction can be accessed by right clicking the new node representing the interaction and selecting BioGateway > Open PubMed Annotations. This will display all the PubMed articles supporting that specific relation. When clicking on one of the articles, a Web Browser window will open redirecting the user to the PubMed page for the selected article.

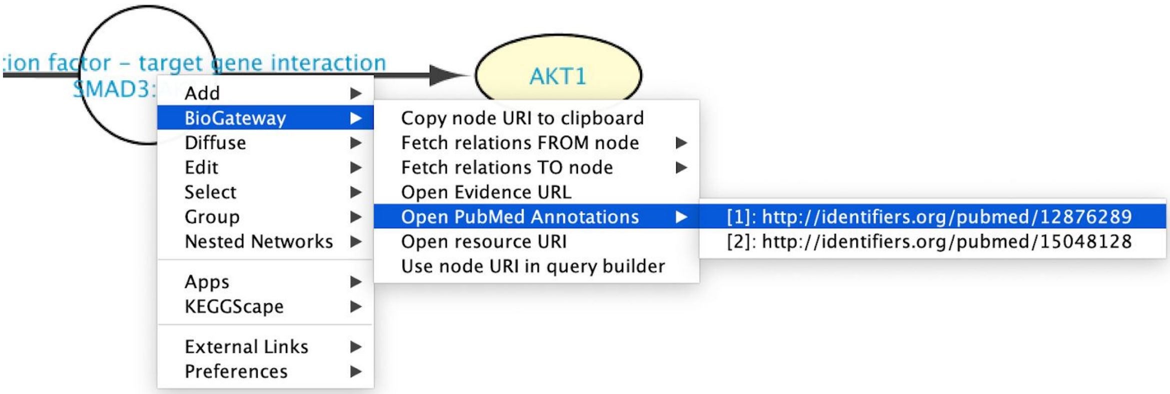

Going to 'Landing Pages'

The Landing Pages contain all the information about each node in BioGateway. A Landing Page for a node can be accessed by right clicking on the node of interest and selecting BioGateway > Open resource URI. This will open a Web Browser window taking the user to the Landing Page for the selected node.

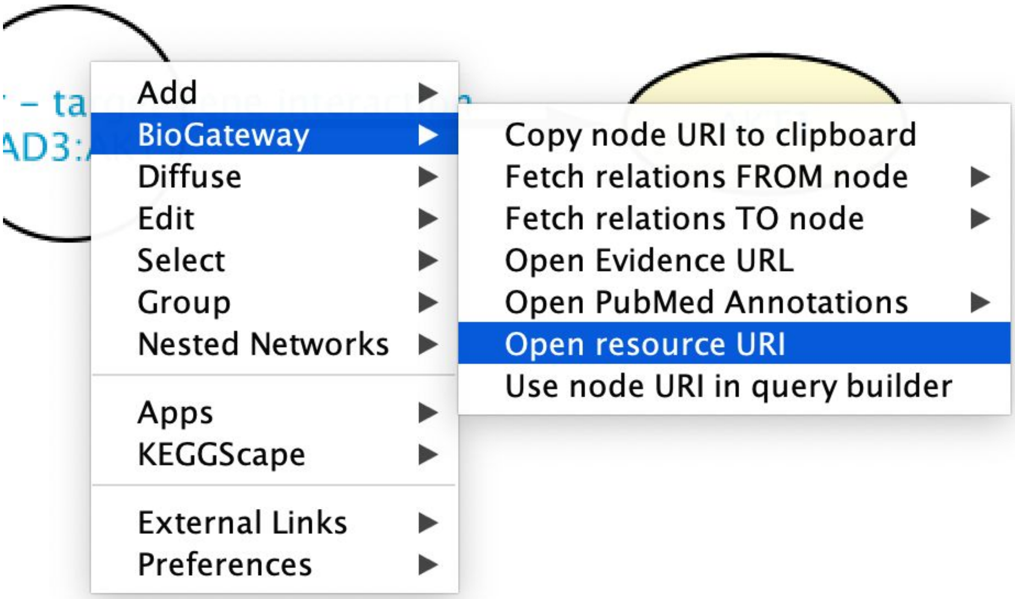

Metadata loading

For performance purposes, the default is to only retrieve a subset of the available information when launching a query. However, the user can select to retrieve more data regarding nodes and relations. This is done by accessing the BioGateway tab in the Cytoscape Control Panel.
In the Active Properties menu that is displayed, Edge and Node Metadata Types branches to expand the different types of metadata that can be loaded. Select the desired metadata, and when completed, click the Reload Metadata button to add the requested information to the network. The information will be now available in the Attribute Table Panel of Cytoscape.
In cases where a network is very large, Metadata loading can take a cumbersome amount of time. If that is the case, the user may abort the action, uncheck some of the Metadata Types and click the Reload Metadatabutton.
Please note that any launched query will import the selected Metadata Types. Thus, it is recommended to make sure to uncheck the Metadata Types before importing any results, specially if the user is about to import a large network.

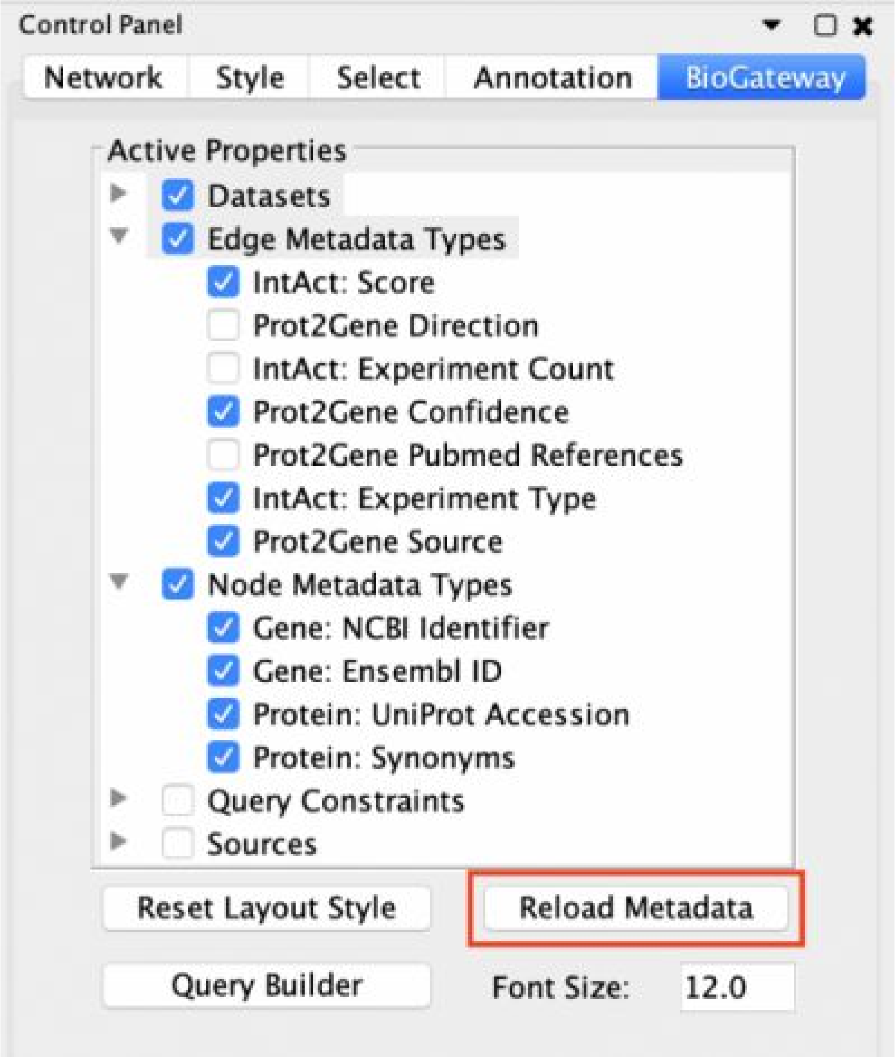

Bulk Query

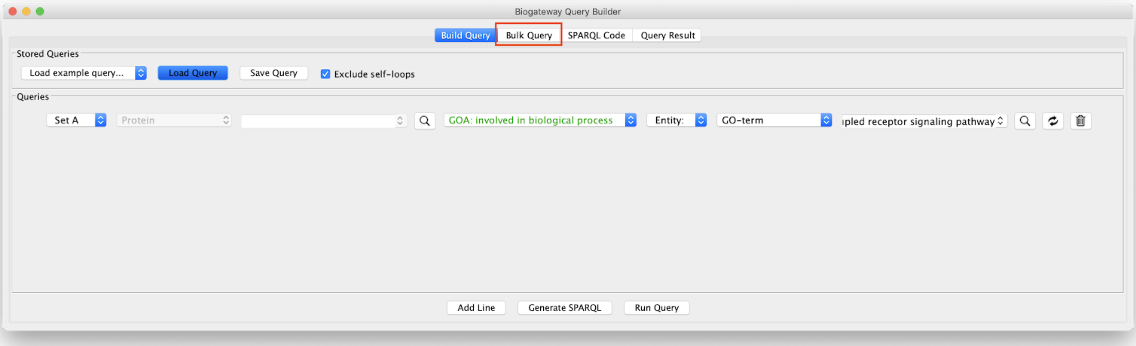

BioGateway also allows to perform explorative network building starting not from a single entity or query line, but by specifying several nodes simultaneously, for instance if a user is interested in extending a network that already was produced through other means in Cytoscape. The BioGateway App therefore supports the possibility of bulk import several nodes from an existing network in the Cytoscape Canvas.
By activating the Bulk Query Import tab in the Query Builder window, the query window below opens.

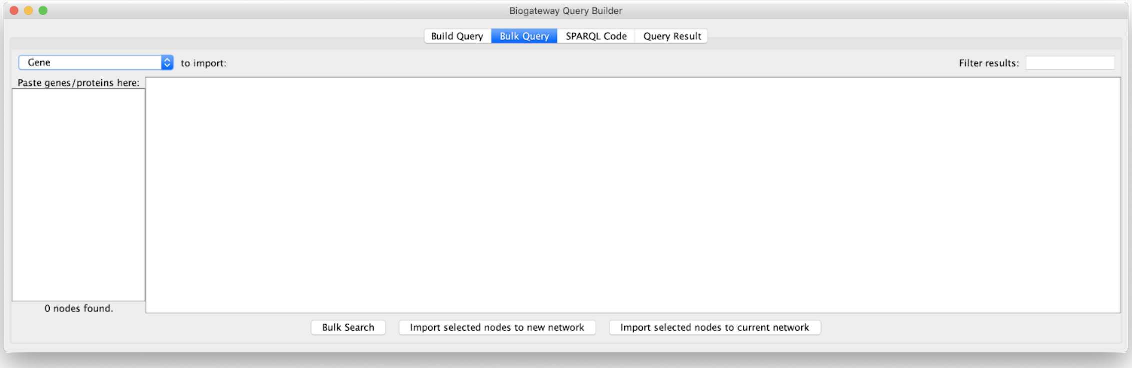

For a bulk query, enter or copy a list of names genes/proteins in the field on the left, each entry separated by a new line. The drop-down menu in the top left corner allows to select the identifier type of the entered entities. Please note that the tool only supports the search of one type of node at a time (Genes or Proteins). Thus, make sure that the desired node type is selected before importing. The supported identifier types are Gene Name, Protein Name, Uniprot Accession, ENSEMBL ID and Entrez ID.
Next, click the Bulk Search button to run the search and display the results. Results can be filtered with the Filter results tool in the top right corner. This tool works exactly in the same way as described in the Query Result section of the Manual.

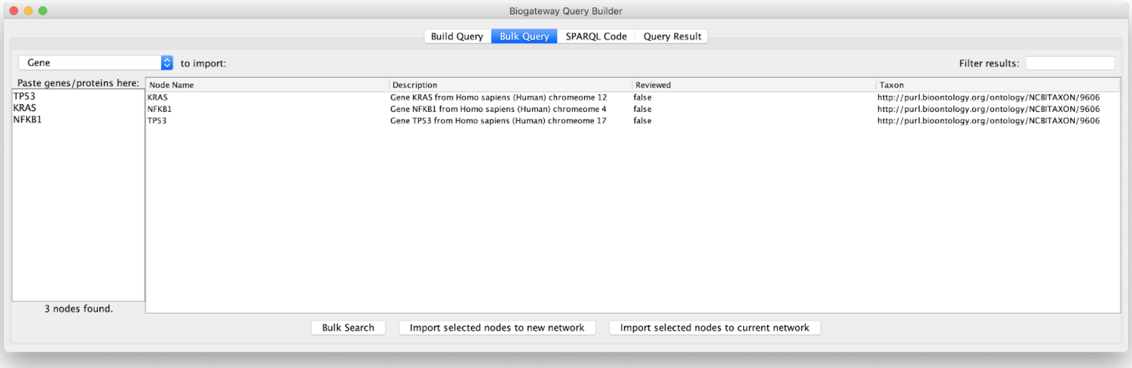

The user can now select the results of interest and load them in a new network or to a current network by clicking the appropriate button below the results window.
It is important to remember that BioGateway represents proteins and genes as two different types of nodes. This means that if the user selects Gene as the node type to import, the results will be imported as genes, while they will be imported as proteins if Protein is selected.

Right Click Queries

Besides using the Query Builder, the user can also run simple queries by right-clicking on a node in the Cytoscape Canvas. This is equivalent to a one line query in the Query Builder, where the Subject/Object would be the node that is being right-clicked. Furthermore, it is also possible to select a group of Nodes and create a query where all the selected nodes are being used as Subject/Object. In the Query Builder, this would be equivalent to a query where each line is representing one of the selected nodes.
The right-click functionality also allows the import of nodes to an empty or existing network. Thus, this tool enables the user to either start from scratch a new network or expand an existing one by exploring the different possible relation types that BioGateway offers.

Adding nodes

To add nodes to an empty or existing network, right-click anywhere in the Cytoscape Canvas and select BioGateway > Add BioGateway node.
This will open a dialog where the user will have to select a node of interest. First, select the type of identifier that should be used, from the possibilities Name, URI, UniProt ID and GO-Term displayed in the top left dropdown menu.
Next, specify the identifier in the text field at the top center of the window. To following step will be to select the type of entity, which is done at the top right dropdown menu. The options in this case are: Protein, Gene, GO-Term, Taxon and Disease.

Once completed, click the Search button to run the query. If the query contains any results they will be displayed in the window. In case of multiple results, the user can filter them by using the Filter results text field in the bottom left corner, or by sorting the columns. The filtering tool and the sorting works in the same way as the filtering tool described in the Query Builder Results section.
Finally, select the node or nodes to import and click the Use Selected Nodebutton to import them into the currently selected Network.

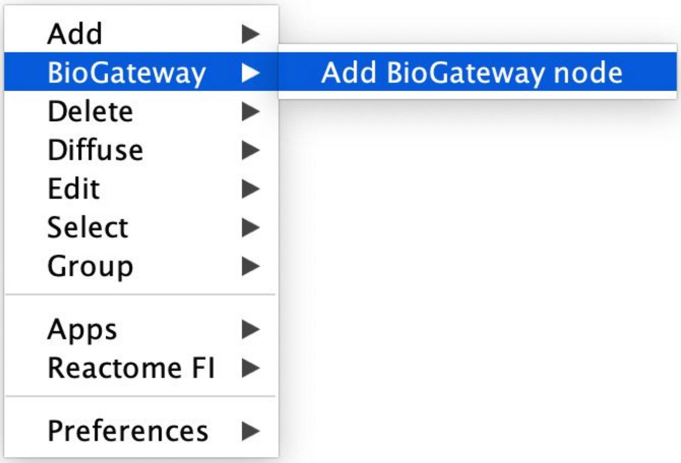

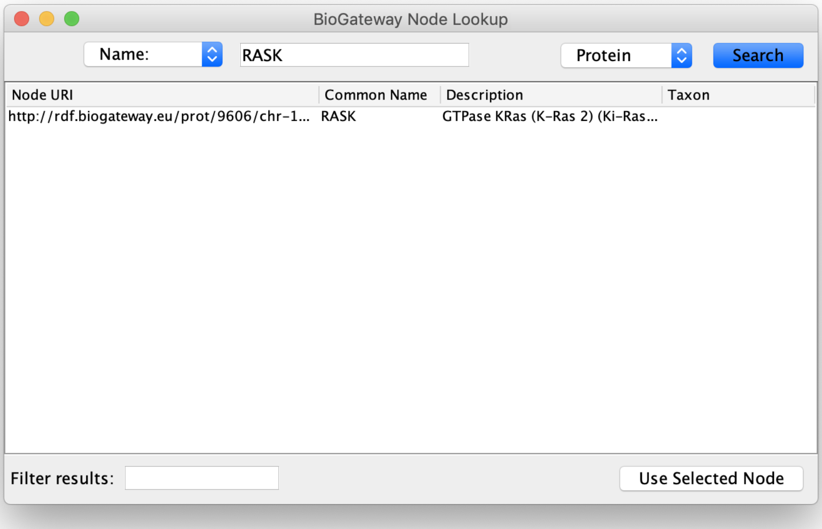

Running queries on the nodes in the network

As mentioned previously, the right-click feature of the BioGateway App allows to quickly run queries using selected node/s as Subject/Object. To run queries on one specific node, right-click the node of interest and select BioGateway > Fetch relations FROM/TO node > select the relation type. If the user wants to use the selected node as Subject, select Fetch relations FROM node. Likewise, if the selected node is the Object of the query, select Fetch relations TO node. The available relation types will depend on the type of node that is being

selected as well as on the selected node being the Subject or the Object of the query. Alternatively, the user can also select Search for all relation types to fetch all possible relations FROM or TO the selected node.
Clicking on one of the possible relations will trigger the query, which will open a new window displaying its results. Here, the user can select all or a subset of the results and import them into the current network by clicking the Import Selected button. BioGateway also offers the possibility to import only the relations from the results that involve any of the nodes present in the current network. This is done by simply clicking the Import relations between existing nodes button.

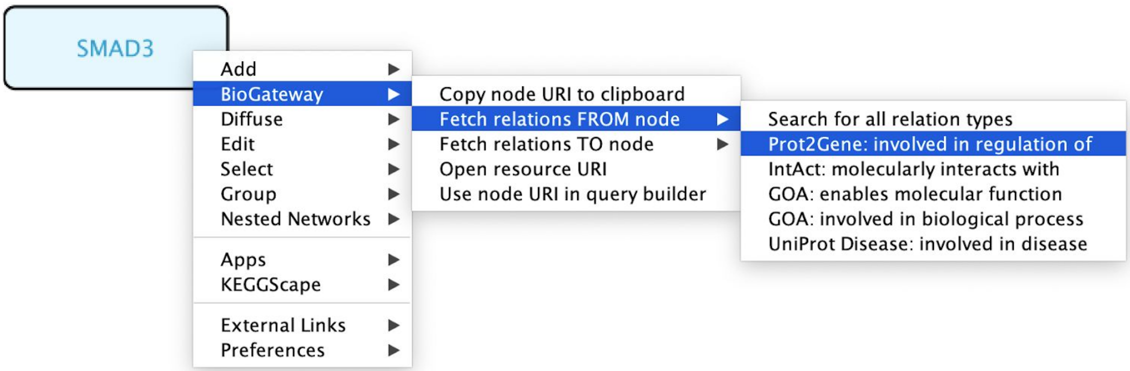

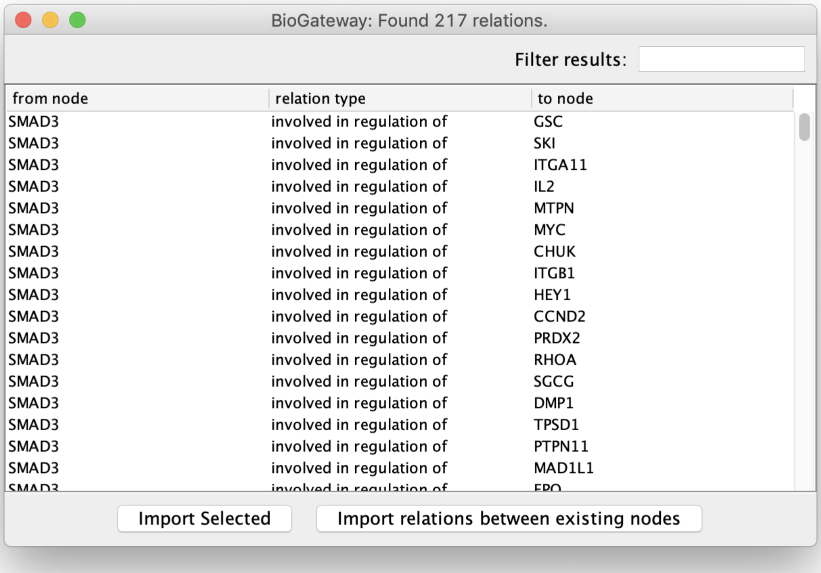

It is also possible to simultaneously use several nodes to create a right-click query. To do so, start by highlighting the nodes of interest followed by right clicking any of them. Now, select BioGateway > Fetch relations FROM/TO selected > select relation type. Please note that when doing this all the nodes will be used as either Subject or Object. After selecting the relation type, the query will be launched and the results will be displayed.

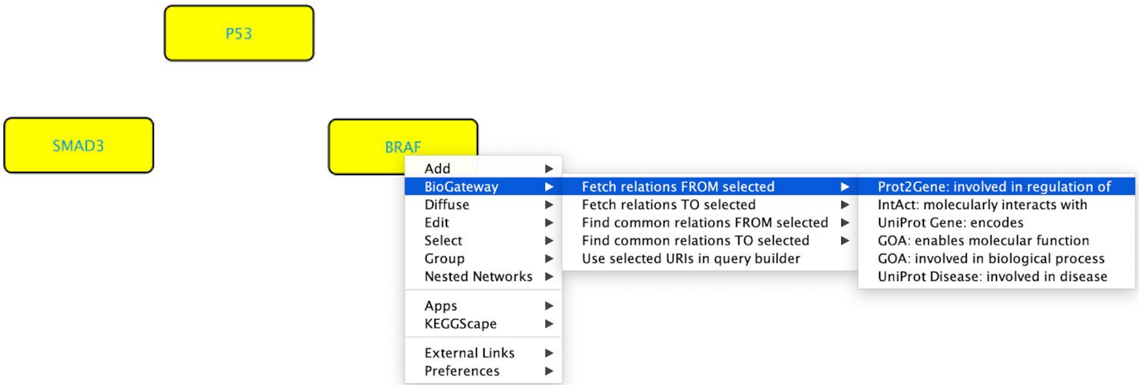

Find common relations FROM/TO selected

When having selected several nodes, it is also possible to search for common relations FROM/TO the selected nodes. This will add a parameter to the search, which will filter only the relations that are shared by a minimum number of the selected nodes.
To get common relations FROM/TO the selected nodes, start by selecting the nodes of interest. Next, right-click any of them and select BioGateway > Find common relations FROM/TO selected > select relation type.
This will trigger a dialog box asking for the minimum number of relations in common going FROM or TO the selected nodes. The user can either enter a number, which will be the parameter used in the filtering of the results, or click the Most in common button. This button will just set the parameter to the highest number of common relations between all the selected nodes. The next step will be to select all the results or only those that are of interest and import them to the current network by clicking the Import Selected button. Alternatively, the Import relations between existing nodesoption can also be used.

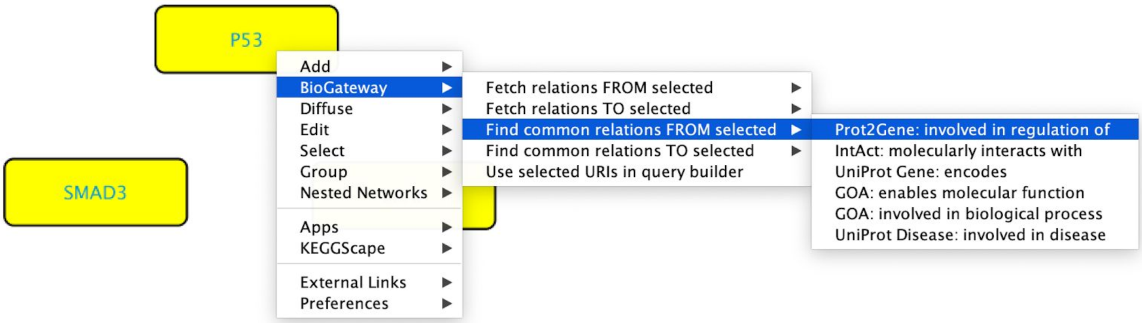

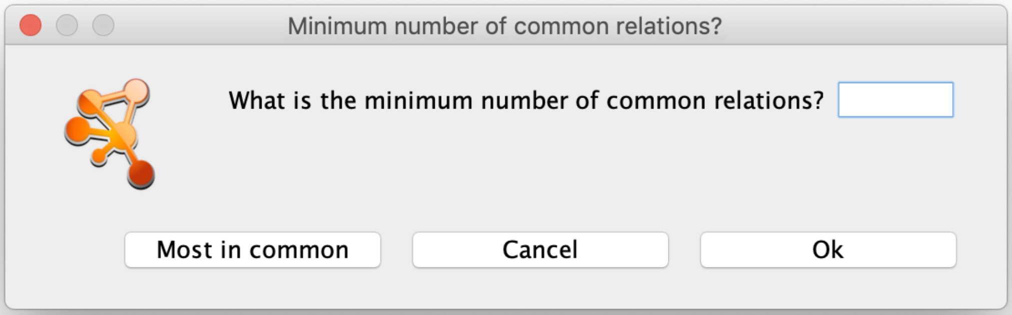

Selecting/deselecting active properties in the Control Panel

In some cases the users may want to work only with some specific types of relations, while disregarding the others. The BioGateway App offers the possibility of enabling or disabling the different relation types that will appear available when right-clicking on one or several nodes and searching for relationships FROM/TO them.
This is done through the BioGateway tab in the Control Panel of Cytoscape, in the Datasets branch of the Active Properties tree.

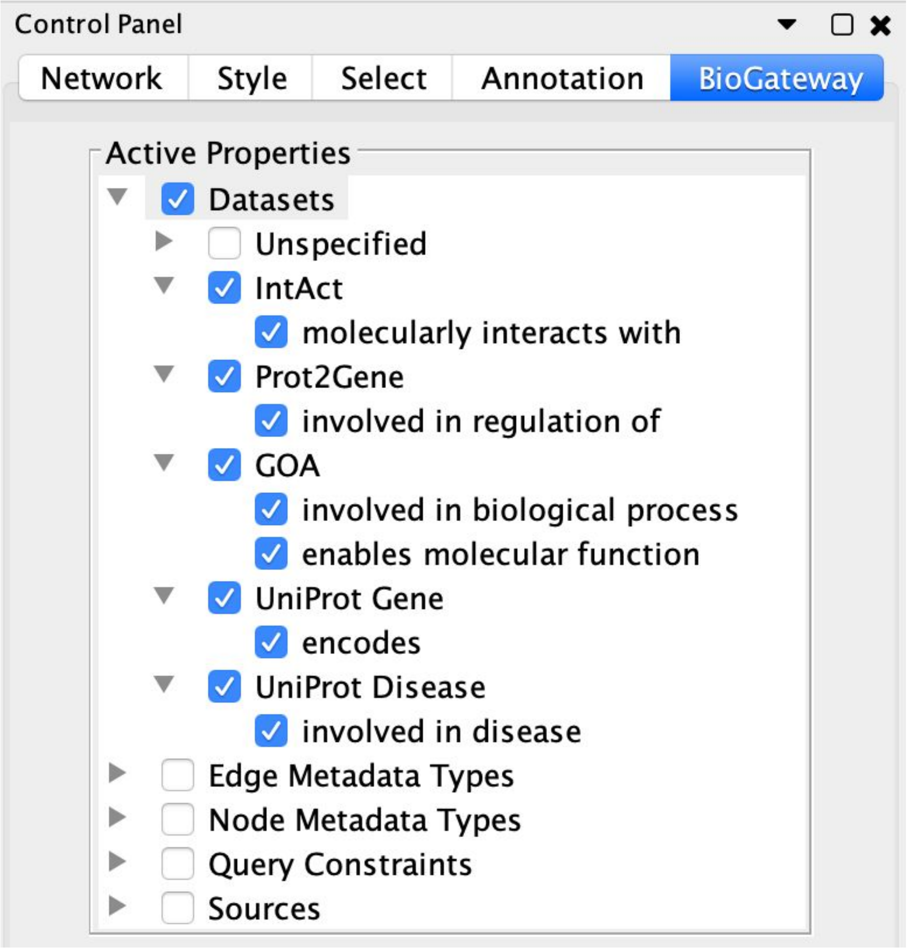

The BioGateway Layout Style

The BioGateway Cytoscape App contains a layout style for the different types of nodes and edges. This layout style is intended to ease the identification of each element in the current network. By default, the BioGateway layout will be automatically applied. If this is not the case, the user can reset the layout style by navigating to the BioGateway Tab in the Cytoscape Control Panel and clicking the Reset Layout Style button. Next, navigate to the Style Tab in the Cytoscape Control Panel, click on the dropdown menu containing the different preloaded styles and select BioGateway.

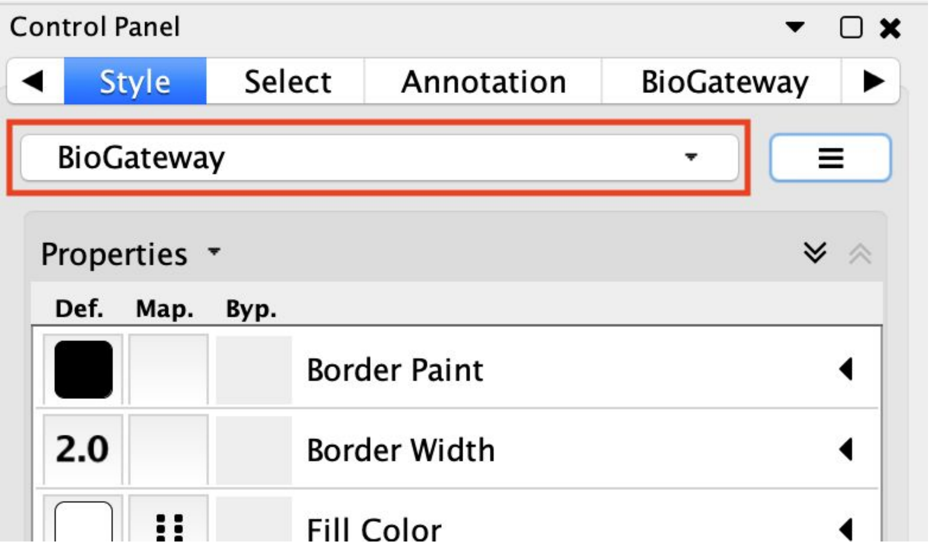

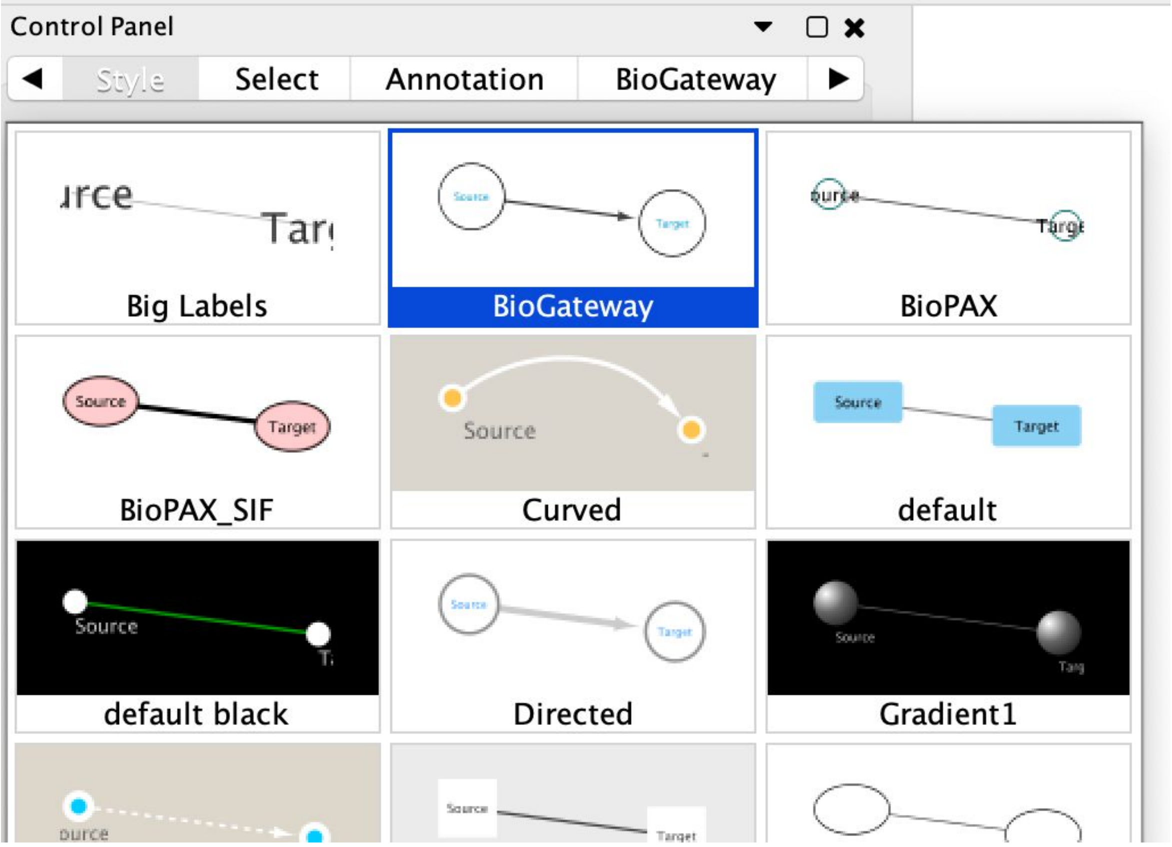

Nodes Layout

There are four layout styles for the nodes. These are:

- Genes: represented by yellow ovals.
- Proteins: represented by blue rounded rectangles.
- Gene Ontology terms: represented by white diamonds.
- Diseases, and other nodes: represented by white circles.

Edges Layout

The different layout possibilities for the edges in BioGateway are:

- Protein-Protein interactions: represented by green, dashed bidirectional arrows. These edges connect two protein nodes.
- Transcription Factor – Target Gene interactions: represented by green, solid unidirectional arrows. This type of edge connects a Protein to a Gene.
- Encoded by: represented by blue, dotted unidirectional arrows. These edges connect a Gene node to a Protein node, representing that Gene A is encoding for Protein B.
- Gene Ontology annotations: represented by dashed, black, unidirectional arrows. This type of edge connects a Protein to a Gene Ontology term.
- Disease annotations: represented by black, dashed unidirectional arrows. These edges connect a Protein to a Disease node.
- Rest of edges: all the other edges are represented by a gray, solid, unidirectional arrow.

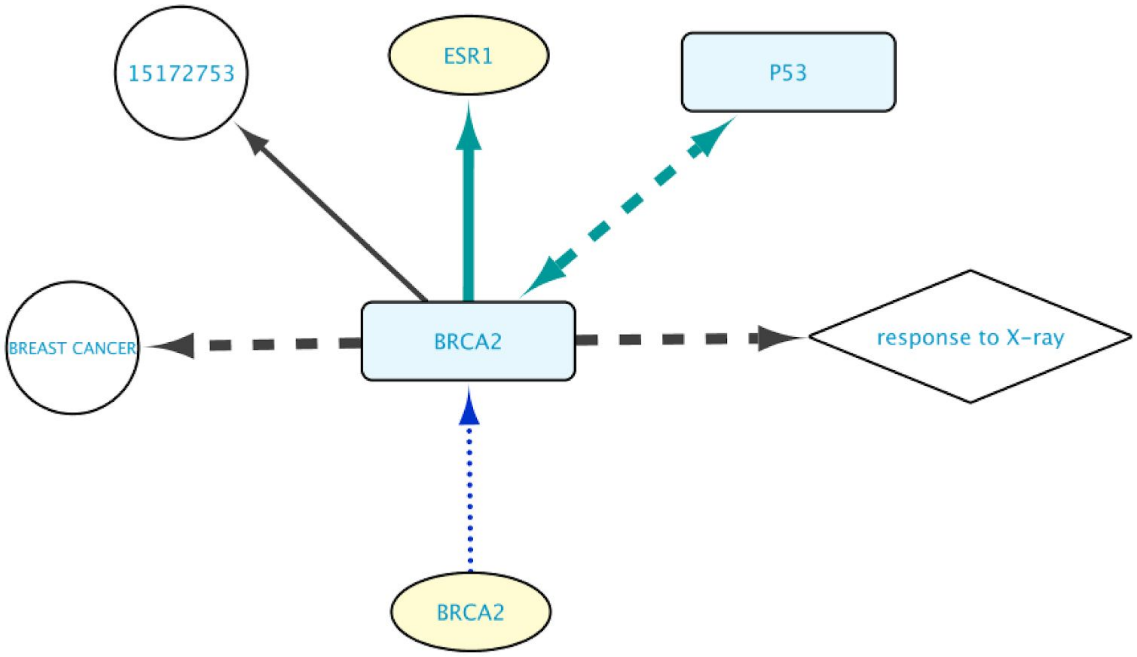

Changing the Font Size in the Query Builder

Depending on the size of the used screen, the Query Builder window can take up a lot of space. To aid the rearrangement of the window, the users can select the font size that will be displayed in the Query Builder.
By default, the font size is set to 12. To change it, simply navigate to the BioGateway tab in the Cytoscape Control Panel and set the desired value.

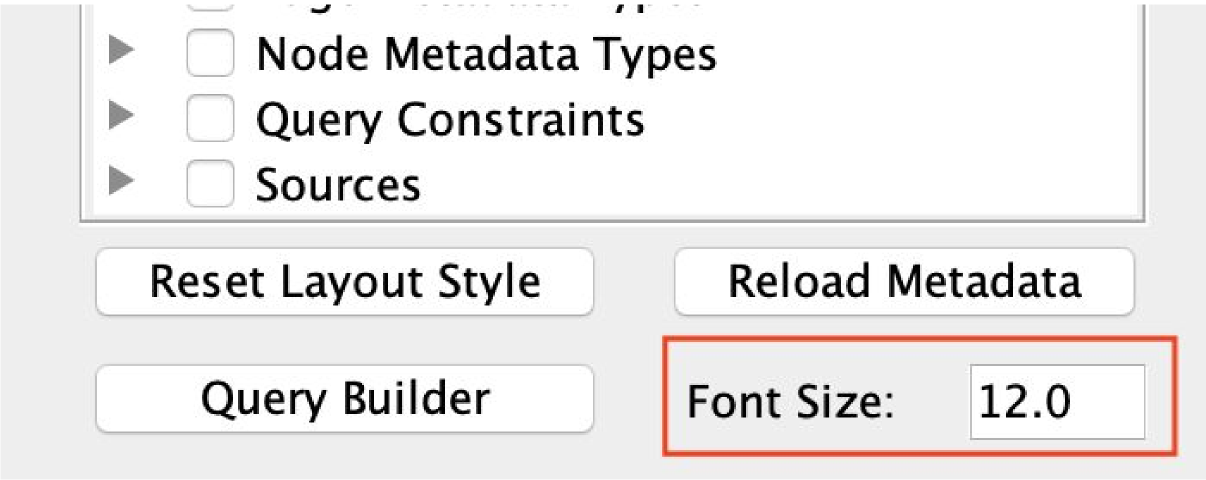
Node Metadata Types
Query Constraints
Sources
Reset Layout Style
Reload Metadata
Query Builder
Font Size:
12.0
