# Supplementary material for: The Cytoscape BioGateway App: explorative network building from an RDF store
Source: Bioinformatics. 2019 Nov 9;36(6):1966–7. doi: 10.1093/bioinformatics/btz835 (PMC7703768; doi:10.1093/bioinformatics/btz835)
Supplement: btz835_Supplementary_Data [file btz835_supplementary_data.zip › btz835-Suppl_Data/BioGatewayApp_SupMat_documentation_S1.docx]

# BioGateway App Supplementary Material S1: Introductory Example

This example illustrates how to use the BioGateway App to retrieve a small network with biological meaning. The main workflow when using the BioGateway App is to start with a biological question, which will then be translated to a network by building a query retrieving the information from the BioGateway server. By adding or deleting lines, the user can create more specific or general queries.

In this case, the example (also available through the Load Query function of the App) illustrates how to retrieve a small network starting with proteins that should have a kinase activity and an involvement in colorectal cancer, including some of their regulatory biological context. This is addressed by a composite query that selects transcription factors, their target genes, the proteins encoded by these genes and their protein interaction partners, and some disease and ontology concepts. Figure S1 displays the main steps to go through to build such a network. The individual query lines (Fig. S1a) define a consecutive series of conditions to be fulfilled by the resulting network. The main information that is subject to the query is obtained from IntAct (protein-protein interactions; Orchard et al. 2013), UniProtKB (Protein descriptions, their genes, related diseases; UniProt Consortium, 2017) and the Gene Ontology database (protein annotations; Gene Ontology Consortium, 2018). To allow a user a special focus on gene regulation we also included several resources with regulatory relations (Transcription Factor - Target Gene) of Transcription Factors (TF) with one or more Target Genes (TG): TFactS (Essaghir et al. 2010), TRRUST (Han et al. 2015), IntAct (Orchard et al. 2013), Signor (Perfetto et al. 2015), HTRIdb (Bovolenta et al. 2012), and GOA (Gene Ontology Consortium, 2018). In addition to these curated resources, we have added a resource produced by a text mining effort, named EXTRI (www.extri.org).


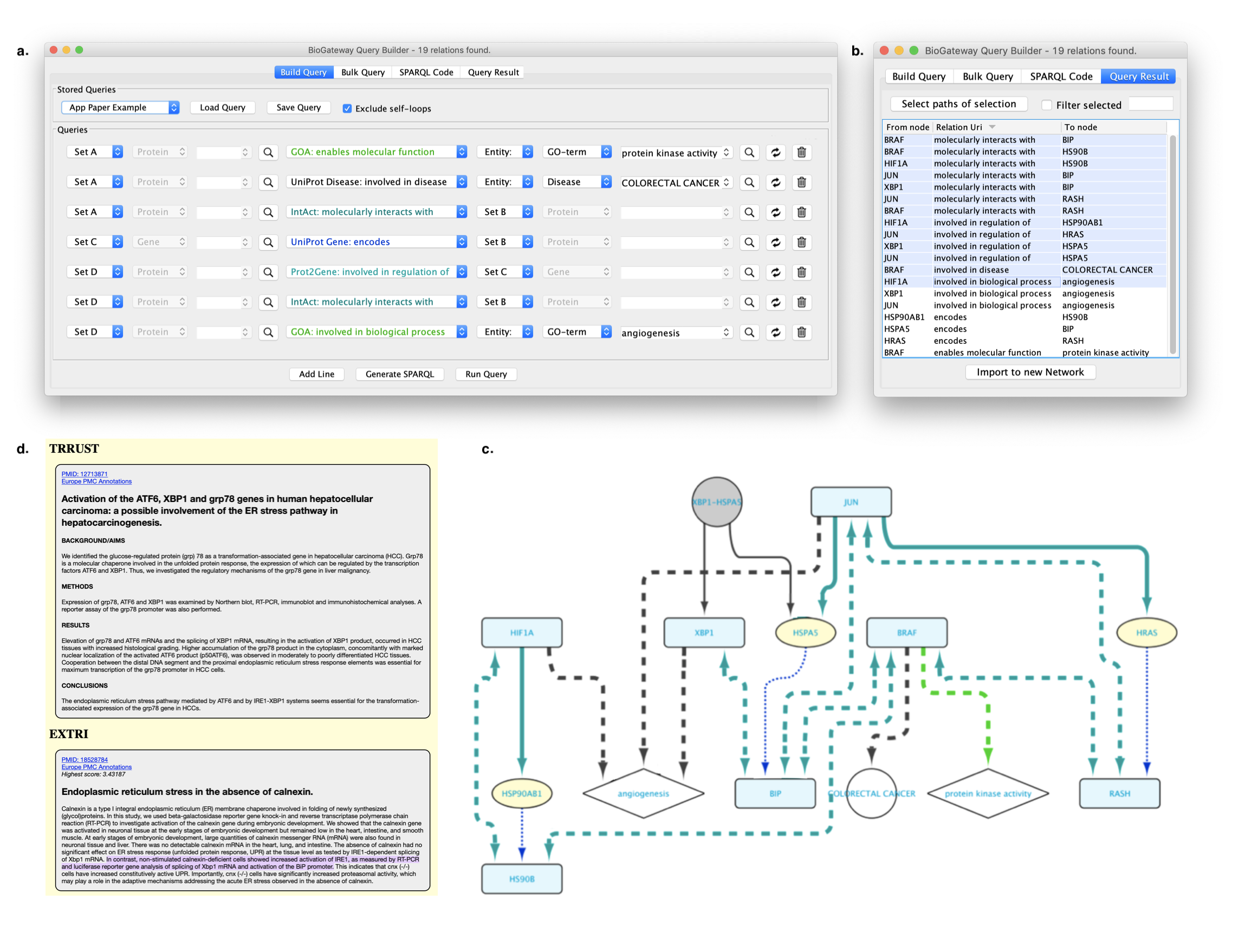


**Fig. S1. Overview of BioGateway App functionality.** Clockwise from top: 1a: query pane with 7 query lines specified; 1b: a selection of the query results (blue part) that are to be displayed as a network; 1c: the network generated from the results with a highlighted TF-TG relation (node in grey); and 1d: Landing page with abstracts and links to papers supporting the TF-TG relationship in 1c. The purple highlight (bottom pane) marks the sentence from the EXTRI resource, identified by text mining.

The sub-parts of the query (Fig S1a, line by line) specify 1) proteins that have a kinase activity; 2) selection of those proteins that have an involvement in colorectal cancer; 3) the identification of their protein-interaction partners; 4) the identification of the genes that code for these protein interaction partners; 5) the transcription factors that regulate the expression of those genes; 6) the selection of the subset of transcription factors that have a protein interaction with any of the proteins found in query line 3; and 7) a further selection of TFs that are involved in angiogenesis. Note that genes need to be linked to their corresponding proteins (line 4) before further relationships in a protein network can be included.

With each query line the *Run Query* command will yield preliminary results for inspection, and if deemed interesting the results can be selected (Fig S1b, Blue field) and displayed as a network in the network pane of Cytoscape (Fig S1c). Note that with each additional query line (the *Add Line* button), the number of results can either be further decreased or increased, until a satisfactory network size is obtained. Useful queries can be stored for future use (*Save Query*). Alternative to building a new query some example queries (*Load example query*) are provided (including the App paper example used here), or queries can also be loaded from file (*Load Query*).

Displaying the results as a hierarchical network (Fig S1c) allow a visual inspection of relationships and regulatory interactions, each indicated by specific edge types, linked to proteins (rounded squares), genes (ovals) and GO terms (diamonds). The network offers not only all the interactive analysis options that Cytoscape supports, but also several functions are added and supported by BioGateway metadata. Double-clicking on a solid arrow extending from the TF XBP1 to gene HSPA5 opens a grey node that provides a link to a ‘landing page’ explaining what the TF-TG interaction is based on: its presence in a curated resource, or the abstract of the paper where the TF-TG was found (Fig S1d). For this specific TF-TG the information is both supported by TRRUST (Fig 1d, top pane) and by the EXTRI resource (bottom pane), where the sentence found by text mining is highlighted. The landing page panes contains links to PubMed and Europe PMC, where a user may further check the validity of e.g. the text mined TF-TG relationship. Additional interaction with the network includes 1) import metadata to display confidence scores as edge weights, and filter/select for that; 2) include metadata concerning the TF-TG interactions and display only interactions supported by selected repositories; 3) expand the network around proteins/genes of interest by right-clicking and local expansion. The application enables many more ways to analyze networks, as explained in the Supplementary material S2 tutorial and on the website ([www.biogateway.eu/app/](http://www.biogateway.eu/app/)).

**References**

Bovolenta, LA. e*t al.* (2012) HTRIdb: an open-access database for experimentally verified human transcriptional regulation interactions. *BMC Genomics*, **13**, 405.

Essaghir, A. e*t al.* (2010) Transcription factor regulation can be accurately predicted from the presence of target gene signatures in microarray gene expression data. *Nucleic Acids Res.*, **38**, e120—e120.

Gene Ontology Consortium (2018) The Gene Ontology resource: 20 years and still GOing strong. *Nucleic Acids Res.*, **47**, D330—D338.

Han, H. e*t al.* (2015) TRRUST: a reference database of human transcriptional regulatory interactions. *Scientific Reports*, **5**, 11432.

Orchard, S. e*t al* (2013) The MIntAct project—IntAct as a common curation platform for 11 molecular interaction databases. *Nucleic Acids Res.*, **42**, D358—D363.

Perfetto, L. e*t al.* (2015) SIGNOR: a database of causal relationships between biological entities. *Nucleic Acids Res.*, **44**, D548—D554.

UniProt Consortium (2017) UniProt: the universal protein knowledgebase. *Nucleic Acids Res.*, **45**, D158—D169.
